# A human telomerase reverse transcriptase-derived peptide GV1001 rescues neurodegeneration in a mouse model of Alzheimer disease

**DOI:** 10.1038/s12276-026-01729-9

**Published:** 2026-06-03

**Authors:** Younghwan Lee, Hyeri Nam, Ji-Won Lee, Yeo Jin Ko, Eum Ji Kim, Sehee Ha, Nan Kim, Ja Wook Koo, Taekwon Son, Sangjae Kim, Seong-Woon Yu

**Affiliations:** 1 Department of Brain Sciences, Daegu Gyeongbuk Institute of Science and Technology (DGIST), Daegu, Republic of Korea; 2 Department of Physiology, School of Medicine, Kyungpook National University, Daegu, Republic of Korea; 3 Emotion, Cognitive & Behavior Research Group, Korea Brain Research Institute (KBRI), Daegu, Republic of Korea; 4 Korea Brain Bank, Korea Brain Research Institute (KBRI), Daegu, Republic of Korea; 5 Teloid Inc., Los Angeles, CA USA

**Keywords:** Alzheimer's disease, Recombinant peptide therapy

## Abstract

GV1001 is a peptide consisting of 16 amino acids derived from the catalytic subunit of human telomerase reverse transcriptase. A recent phase II clinical trial in patients with Alzheimer disease (AD) showed that GV1001 effectively improved memory impairment with proven safety, leading to larger clinical trials. However, the mechanisms underlying therapeutic effects of GV1001 on AD remain elusive. Here, we report that GV1001 reduces amyloid plaque burden and rescues synaptic loss and memory deficits in 5xFAD mice by increasing microglial migration toward large amyloid plaques and amyloid β degradation. Single-cell RNA-sequencing revealed that GV1001 promoted the migratory and phagocytic phenotypes by modulating disease-associated microglial profiles. At the molecular level, through virtual target screening and docking simulation combined with peptide pulldown, we identified that bradykinin receptor 1 is the binding target of GV1001. Furthermore, we revealed that GV1001 facilitated microglial migration and amyloid β phagocytosis in an mTORC2-dependent manner. Collectively, our work demonstrates the amyloidolytic effects and the relevant in-depth signaling mechanism of GV1001 in microglia, suggesting GV1001 as a promising disease-modifying therapeutic agent for AD.

## New insights on GV1001’s role in Alzheimer’s treatment

Alzheimer disease (AD) is marked by amyloid β accumulation and neuroinflammation, with microglia having a dual role in disease progression. This study explores GV1001, a peptide initially developed for cancer, which has shown promise in AD treatment. Researchers used the 5xFAD mouse model to investigate the effects of GV1001, finding that it reduces amyloid burden by enhancing microglial migration and Aβ clearance. GV1001 binds to bradykinin receptor 1, activating the mTORC2 pathway, crucial for microglial migration and phagocytosis. This activation leads to improved memory and synaptic integrity in mice. The study highlights GV1001’s potential as a therapeutic agent for AD, emphasizing its role in modulating neurodegenerative microglial phenotypes. Future research could further elucidate GV1001’s mechanisms and explore its effects on other neural cells, potentially broadening its therapeutic applications. This summary was initially drafted using artificial intelligence, then revised and fact-checked by the author.

## Introduction

Alzheimer disease (AD) is the most common age-associated neurodegenerative disease pathologically defined by accumulation of aggregated amyloid β (Aβ) and intracellular neurofibrillary tangles^1^. According to the amyloid hypothesis, overproduction and accumulation of Aβ have key roles in the pathogenesis of AD, which is accompanied by synaptic and neuronal loss and progressive cognitive decline^2^. In addition, overt microglial activation and neuroinflammation in response to Aβ accumulation contribute to neurodegeneration in AD^3^.

Several genome-wide association analyses have brought microglia into the center of attention by revealing that many genetic variants increasing risk of AD are primarily expressed in microglia^4–6^. Microglia are principal immune cells resident in the central nervous system and are able to phagocytose Aβ and lower Aβ burden^7^. Also, microglia engagement with Aβ deposits and formation of the microglial barrier can serve protective roles by compacting Aβ plaques and limiting the propagation of Aβ accumulation^8^. Therefore, microglia response may be protective against the onset of AD. However, pro-inflammatory activation suppresses microglial phagocytosis of Aβ, impairing Aβ clearance^9^. In response to Aβ accumulation, microglia progressively acquire pro-inflammatory phenotypes and overproduce neurotoxic inflammatory mediators, exacerbating neurodegeneration^10,11^. Therefore, microglia activation can yield both beneficial and detrimental outcomes. These complex roles of microglia may be reflected by their wide range of different activation states; recent extensive genomics studies at the single-cell or nucleus level have revealed the heterogeneity of microglia phenotypes and identified various subgroups of activated microglia during neurodegeneration^12–14^. As altered microglia function and neuroinflammation are critical components of AD pathogenesis, it is imperative to search for novel therapeutic agents that can restore normal microglia function.

GV1001 is a peptide composed of 16 amino acids derived from the catalytic subunit of human telomerase reverse transcriptase. The original purpose of developing this peptide was for cancer immunotherapy because most malignant tumors achieve their immortality through upregulation of telomerase^15^. GV1001 is safe and indeed induces immune responses with anticancer efficacies in diverse solid cancers such as pancreatic cancer, non-small-cell lung cancer, melanoma, and hepatocellular carcinoma^16–19^. Interestingly, a recent phase II clinical trial of GV1001 showed improved cognition in patients with AD. In patients with moderate-to-severe AD, subcutaneous injection of GV1001 at a dosage of 1.12 mg every week for 4 weeks (4 injections) followed by every 2 weeks until week 24 (10 injections) significantly reduced the decrease in Severe Impairment Battery score compared with the placebo control group, suggesting the beneficial effect of GV1001 (ref. ^20^). GV1001 was well tolerated without notable safety concerns during the trial^20^. Encouraged by these promising results, larger clinical studies of GV1001 for AD are currently in progress: a phase III clinical trial in South Korea (NCT05303701) and a phase II clinical trial in the USA (NCT05189210).

These studies suggest that GV1001 could provide a promising treatment strategy for AD. However, the mode of action underlying the therapeutic effects of GV1001 against AD, especially about microglia profile, is not well known yet. Here, we report the disease-modifying mechanism of GV1001 in the 5xFAD amyloidogenic mouse model. GV1001 reduced amyloid burden by increasing microglia migration toward the large Aβ plaques and promoting Aβ clearance. By combining virtual target screening and docking simulation with peptide pulldown assay, we found that GV1001 bound to bradykinin receptor 1 (B1R). Further study revealed that binding of GV1001 to B1R activated mammalian target of rapamycin complex 2 (mTORC2)-dependent signaling in microglia, suggesting that rescue of neurodegeneration by GV1001 is mainly via microglial B1R–mTORC2 axis. Additionally, our single-cell RNA-sequencing (scRNA-seq) reinforced these findings and found that GV1001 modulated microglial neurodegenerative phenotype.

## Materials and methods

### Mice

All procedures and ethical regulations for the care and use of laboratory animals were approved by and in accordance with the guidelines provided by the IACUC of DGIST (DGIST-IACUC-21051804-0001). Mice were housed in a specific pathogen-free environment under a 12:12 h light:dark cycle at the DGIST animal facility. Heterozygous 5xFAD male mice and C57BL/6 wild-type (WT) littermates were used for all assays. The 5xFAD genotype was confirmed by PCR amplification of tail DNA.

### Cell culture

Primary microglia and astrocytes were obtained from 1–3-day-old postnatal mice as described previously^10^ and cultured in Dulbecco’s modified Eagle’s medium (DMEM, Corning) supplemented with 10% heat-inactivated fetal bovine serum (FBS, Hyclone) and 1% penicillin–streptomycin (Hyclone). Microglia were isolated at days in vitro 12 by tapping.

### Microglia isolation from adult brains

The protocol was modified from the previous study^10^. Brains were dissociated in the buffer containing 20 U/ml DNase I (Stem Cell Technologies), 0.05% collagenase D (Roche) in Hank’s balanced salt solution (Gibco) containing 5 mM CaCl_2_, 0.5% glucose, 15 mM HEPES, pH 7.2 for 20 min at 37 °C with gentle pipetting. The pellets were suspended in Percoll (Sigma-Aldrich) gradient (70%, 37%, and 30% Percoll in Hank’s balanced salt solution). The cell pellets from interphase were resuspended in MACS buffer containing CD16/CD32 antibody for FC-γ receptor blocking (BD Pharmingen, 553142). After 10 min, cell suspensions were incubated with anti-CD11b microbeads (Miltenyi Biotec) at 4 °C for 10–20 min. CD11b^+^ microglia were isolated by using an MACS LS column (Miltenyi Biotec). For lamellipodium study, isolated microglia were incubated in DMEM/F12 (Corning) supplemented with 2.5 mM L-glutamine, 15 mM HEPES, pH 7.2, 10% FBS, and 1% penicillin–streptomycin for 2 days.

### GV1001 administration

For in vitro study, GV1001 was dissolved in PBS. For in vivo study, GV1001 was dissolved in 0.9% sodium chloride (saline) and subcutaneously injected to a final concentration of 1 mg/kg, three times a week for 8 weeks.

### Inhibitors

For treatment of microglia with rapamycin, Torin1, naltrindole, R715, and HOE140, microglia were pretreated with the inhibitors for 30 min before GV1001 treatment. Cytochalasin D was co-administrated for past 2 h during GV1001 treatment. For in vivo study, R715 was intranasally injected to a final concentration of 0.5 mg/kg, five times a week for 8 weeks.

### Generation of Aβ1-42 fibrils (fAβ1-42) and Aβ uptake assay

Monomeric fluorescein isothiocyanate (FITC)-Aβ1-42 (Bachem) and TAMRA-Aβ1-42 (AnaSpec) were dissolved in dimethyl sulfoxide to a stock concentration of 0.5 mM. Then, monomeric FITC-Aβ1-42 or TAMRA-Aβ1-42 was diluted in DMEM containing 10% FBS. Tenfold dilution Aβ1-42 was incubated at 37 °C for 16 h to generate fibrillar form of Aβ1-42 (fAβ1-42). Primary microglia were incubated with TAMRA-fAβ1-42 or FITC-fAβ1-42 (0.3 μM) for 2 h for Aβ uptake assay. After washing to remove the remaining fAβ1-42, images were obtained using an LSM700 confocal microscope.

### Transwell migration assay

Transwell migration assays were performed using 8-μm-pore-diameter inserts (Corning). Primary microglia were plated in the upper chamber, and the chamber was then placed within the bottom wells of a 24-well plate containing medium supplemented or not with drugs followed by incubation for 24 h. The non-migrating cells in the upper chamber were removed with a cotton swab, and the cells on the lower surface were fixed with 4% paraformaldehyde for 15 min and stained with 0.2% crystal violet at room temperature for 30 min.

### Microglial transduction

sh*Bdkrb1* was purchased from Sigma-Aldrich. sh*Bdkrb1* was cloned into the PLKO.1-EGFP vector to generate lentiviruses. Lenti-X 293T cells (Clontech) were transfected with the transfer vector (PLKO.1-sh*Bdkrb1*-EGFP or PLKO.1-shscramble-EGFP), packaging vector (psPAX2, Addgene), and VSV-G envelope-expressing vector (PMD2.G, Addgene) for production of lentiviruses. After 3 days, the viruses in supernatant were harvested using ultracentrifugation (Beckman Coulter). Primary cultured microglia were infected with the lentiviruses in hexadimethrine-bromide-treated medium for 24 h. EGFP expression was monitored after 72 h to estimate the lentiviral transduction efficiency.

### Virtual target simulation

GV1001(EARPALLTSRLRFIPK) was converted to SMILES format and used as input in SwissSimilarity. We chose 2D&3D combined ChEMBL^21,22^ and GLASS^23^ provided in SwissSimilarity as database. After screening, we listed up molecules which showed high similarity (target score ≥ 0.9) with GV1001 and chose top 10 binding candidates for further analysis. For peptide–protein docking simulation, we retrieved human G protein-coupled receptor (GPCR) structure from GPCRdb^24^. GV1001 sequence and GPCR structures were simulated with CABS-dock^25,26^. Obtained peptide–protein docking results were visualized and analyzed with PyMOL and protein–ligand interaction profiler^27^.

### scRNA-seq

For cell dissociation, one cortical slice per mouse was collected and incubated at 37 °C for 30 min in dissociation media (Hibernate-A medium without calcium and magnesium, containing 1 mg/ml papain, 50% D-(+)-trehalose dihydrate, 25 mM DL-2-amino-5-phosphonopentanoic acid, and DNase I) with shaking. After two washes with Hibernate-A medium, the tissues were mechanically triturated in 1.5 ml Hibernate-A medium using pipette tips (2 mm and 1 mm diameters) for dissociation. The suspension was filtered through a 70 μm cell strainer, loaded onto a gradient medium (Hibernate-A medium containing ovomucoid inhibitor-albumin), and centrifuged at 160×*g* for 6 min at 4 °C to remove debris. Dead cells were eliminated using the Dead Cell Removal Kit (Miltenyi Biotec, 130-090-101), according to manufacturer’s instructions. Cell numbers were quantified using the SOL COUNT Automatic Cell Counter, yielding 2,900–21,000 cells/μl across samples. For scRNA-seq, cells were diluted to 1,100–1,800 cells/μl following the 10× Genomics protocol, targeting 10,000 cells for recovery. Single cells were captured using the 10× Chromium platform and processed with the Chromium Single Cell 3’ Reagent Kit v3.1 (Dual Index). Gel Beads-in-Emulsion were generated, and barcoded cDNA was synthesized and purified using silane magnetic beads. Amplified cDNA was used to construct sequencing libraries, with final concentrations ranging from 16.3 ng to 36.1 ng.

### Quantification and statistical analysis

To avoid the influence of gender, male mice were used for all experiments with their littermate WT controls. Mice were randomly divided into each group. A sensible sample size was with the range found in similar methodologies in our previously published literatures and others to verify sufficient reproducibility of the results. For behavioral outcomes, we used a minimum of 18 mice per group. Behavior and immunohistochemistry experiments were conducted in a blinded manner. Gene expression and western blotting analysis were performed unblinded. All in vitro experiments were performed at least three times, each with at least two repeats per experiment. The data were presented as mean ± standard error of the mean (SEM) values. Statistical analysis was performed using an unpaired *t* test or one-way analysis of variance followed by appropriate post hoc tests (parametric and non-parametric) as applicable, and statistical significance was evaluated using GraphPad Prism (GraphPad Software).

### Other methods

Additional experimental methods are provided in the Supplementary material.

## Results

### GV1001 improves learning and memory deficits in 5xFAD mice

**Fig. 1:**
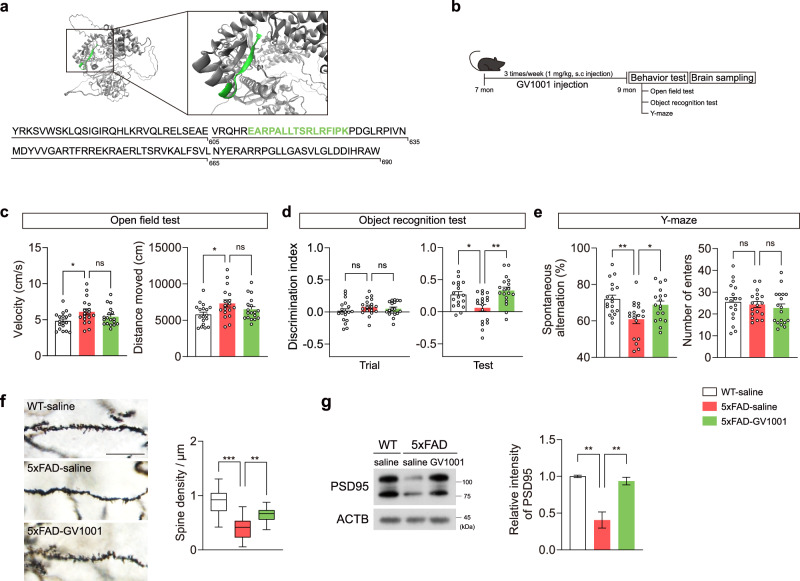
GV1001 rescues memory deficits and synaptic degeneration in 5xFAD mice. **a** Amino acid sequence of GV1001, a peptide derived from human telomerase reverse transcriptase. GV1001 sequence is shown in green. The secondary structure of human telomerase reverse transcriptase was drawn using visual molecular dynamics and AlphaFold prediction^93^. **b** Diagram of the experimental schedule. Open-field test (part **c**) for locomotion analysis, and object recognition test (part **d**) and Y-maze test (part **e**) for memory assessment in wild-type (WT) and 5xFAD mice (*n* = 18 mice per group) after GV1001 injection. **f** Representative images of Golgi–Cox staining (left) for the analysis of spine density in the hippocampal neurons of WT and 5xFAD mice after GV1001 injection. Scale bar, 10 μm. Quantification of the average relative spine density of hippocampal neurons (right; *n* = 4 mice per group). **g** Representative western blots of postsynaptic density protein 95 (PSD95) (left) in the hippocampus from 8–9-month-old WT and 5xFAD mice after GV1001 injection. Mean PSD95 protein levels after normalization to ACTB (right; *n* = 3 per group). All statistical comparisons were conducted with one-way analysis of variance followed by a post hoc test (parametric and non-parametric) as per applicable, *P < 0.05, **P < 0.01, and ***P < 0.001; ns, not significant. Data are means ± SEM. s.c., subcutaneous.

GV1001 is a peptide corresponding to the human telomerase reverse transcriptase sequence at positions 611–626, EARPALLTSRLRFIPK (Fig. 1a). For clinical trials of AD treatments, penetration of the blood–brain barrier (BBB) is important and previous study reported the BBB penetration of GV1001 using MRI^28^. To confirm in a simpler way than MRI that GV1001 can cross BBB and reach the brain, we injected Fe-conjugated GV1001 and detected iron residue by Prussian blue staining. We performed this assay in WT mice, as many AD mice models already suffer from BBB dysfunction^29^. In this assay, we observed positive blue signals in the WT brain, indicating that GV1001 penetrated the intact BBB (Supplementary Fig. 1). To test the effects of GV1001 in AD model mice, we injected 6–7-month-old 5xFAD mice with GV1001 (1 mg/kg) three times a week for 2 months. After the injection, we evaluated the effects of GV1001 on learning and memory (Fig. 1b). Previous studies reported hyperlocomotion of 5xFAD mice in open-field test (OFT)^30^. Similarly, 5xFAD mice showed abnormal hyperlocomotion in OFT, and GV1001 injection did not affect locomotion of 5xFAD mice (Fig. 1c). To examine recognition memory, we conducted novel object recognition test after GV1001 injection. 5xFAD mice showed memory deficits as reflected by a lower interest in a new object, but GV1001 treatment significantly improved recognition memory (Fig. 1d). To assess spatial memory, we performed Y-maze assay. 5xFAD mice showed significantly lower arm alternation in Y-maze assay, but GV1001 treatment effectively recovered these memory deficits (Fig. 1e). Progression of AD is accompanied by synaptic loss, which causes learning and memory impairment. Measurement of spine density using Golgi–Cox staining showed that GV1001 significantly rescued synaptic loss of hippocampal CA1 neurons in 5xFAD mice (Fig. 1f). GV1001 also rescued synaptic strength in 5xFAD mice, as revealed by an increase in the intensity of postsynaptic density protein 95 (Fig. 1g). These data indicate that GV1001 rescues memory decline and hippocampal synaptic loss in 5xFAD mice.

### GV1001 diminishes Aβ plaque burden and neuroinflammation in 5xFAD mice

**Fig. 2:**
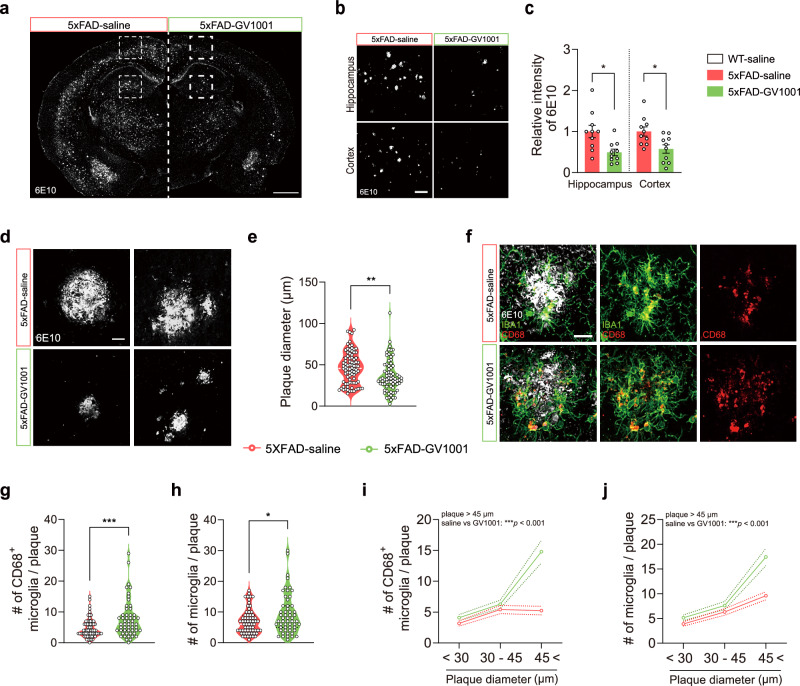
GV1001 reduces Aβ plaque burden by promoting microglia clearance in 5xFAD mice. **a** Representative immunofluorescence images obtained by staining with 6E10 antibody in brains of 8–9-month-old 5xFAD mice injected with saline (left) and GV1001 (right). Scale bar, 1 mm. **b** Enlarged images represent the hippocampus (up) and cortex (down). Scale bar, 100 μm. **c** Quantification of 6E10 intensity in the hippocampus (left) and cortex (right; *n* = 10 mice per group). **d** Representative amyloid β (Aβ) plaque images obtained by immunohistochemistry with 6E10 antibody. Scale bar, 20 μm. **e** Quantification of plaque diameter between 5xFAD-saline and 5xFAD-GV1001 mice group (*n* = 11 mice per group). **f** Representative immunofluorescence images obtained by co-staining with 6E10 (Aβ plaques), IBA1 (microglia), and CD68 (phagocytic microglia) antibodies in the hippocampus of 8–9-month-old 5xFAD mice (*n* = 11 mice per group). Scale bar, 20 μm. Numbers of CD68^+^ phagocytic microglia (part **g**) and total microglia (part **h**) per Aβ plaque. Analysis of the number of CD68^+^ phagocytic microglia (part **i**) and total microglia (part **j**) per plaque depending on the size of Aβ plaques (*n* = 11 mice per group). All statistical comparisons were conducted with unpaired *t* test. *P < 0.05, **P < 0.01, and ***P < 0.001. Data are means ± SEM. WT, wild-type.

Next, we examined whether the memory improvement effect of GV1001 is associated with reduction of Aβ plaque burden. Immunohistochemical analyses with 6E10 antibody revealed that GV1001 treatment remarkably reduced the intensity of Aβ plaques in both the hippocampus and cortex, compared with 5xFAD-saline mice (Fig. 2a−c). The presence of Aβ plaques is accompanied by robust neuroinflammation, as evidenced by increases in IBA1 and GFAP intensities^31^. As expected, GV1001 significantly decreased IBA1 and GFAP intensities in the 5xFAD hippocampus and cortex (Supplementary Fig. 2a−d). Furthermore, the transcript levels of representative cytokines (*Il1b*, *Il6*, *Tnf*, and *Cxcl1*) were significantly downregulated both in the hippocampus (Supplementary Fig. 2e) and in the cortex (Supplementary Fig. 2f) following GV1001 injection. These data indicate that GV1001 injection effectively decreased amyloid plaque burden and attenuated neuroinflammation in 5xFAD mice.

### GV1001 increases phagocytic microglia around the large Aβ plaques in 5xFAD mice

Consistent with a reduction in Aβ plaque burden, GV1001 significantly decreased the average size of Aβ plaques (Fig. 2d, e). Because reduction in amyloid pathology may occur through decrease in the production of Aβ peptides or increase in the clearance of Aβ plaques, we assessed amyloidogenesis. Amyloidogenic processes of full-length APP (APP-FL) for Aβ production are mediated through sequential cleavages by β-secretase (BACE) and ɣ-secretase complexes, and alteration of this processing induces Aβ accumulation and aggregation in the brain^32^. To examine whether the reduction of Aβ plaque burden by GV1001 is associated with alteration in amyloidogenic processing, we examined the key factors involved in Aβ production. Although APP-FL level was slightly decreased in the hippocampus of GV1001-injected 5xFAD mice, other factors — APP C-terminal fragment (APP-CTF), soluble APP-alpha (sAPPα), BACE1, components of ɣ-secretase complex, presenilin (PSEN) 1 and 2 — did not significantly change in comparison with 5xFAD-saline group (Supplementary Fig. 3a,b). GV1001 affected none of the factors, including APP-FL, in the cortex of 5xFAD mice (Supplementary Fig. 3a,c).

Because GV1001 did not alter amyloidogenic APP processing of 5xFAD, we wondered whether GV1001 promotes microglial clearance of Aβ plaques. To that end, we examined whether GV1001 would increase the number of phagocytic microglia by using an antibody against CD68, a marker for the late endosomes and lysosomes. Importantly, GV1001 increased the number of CD68-positive phagocytic microglia near the plaques (Fig. 2f, g). Of interest, the total number of microglia around each Aβ plaque was also much higher in GV1001-injected 5xFAD mice than in the saline-injected group (Fig. 2h). When the number of plaque-associated microglia was analyzed depending on plaque diameter, the increases in the number of both CD68^+^ and total microglia by GV1001 were associated mostly with large plaques (diameter> 45 μm; Fig. 2i, j). By contrast, astrocyte recruitment toward Aβ plaques was not induced by GV1001 (Supplementary Fig. 4a−c), suggesting that GV1001 exerts its amyloidolytic effect mainly through microglia. Overall, these data suggest that GV1001 ameliorates amyloid pathology by recruiting phagocytic microglia targeting large Aβ plaques.

To elucidate whether the increased number of microglia was due to an increase in proliferation, we measured the number of KI67 (marker of proliferation)-positive microglia near the plaques and found that it did not differ between GV1001-injected and saline-injected 5xFAD groups (Supplementary Fig. 5a,b). We also examined whether GV1001 increases the number of microglia by decreasing cell death of plaque-associated microglia. We performed terminal deoxynucleotidyl transferase dUTP nick-end labeling (TUNEL) assay and co-staining with IBA1 to detect cell death in microglia and found no differences in the cell death rates of microglia around Aβ plaques between GV1001-injected and saline-injected 5xFAD (Supplementary Fig. 5c,d). These results indicated that the GV1001 increased the number of microglia around plaques mainly by promoting microglia recruitment toward Aβ plaques.

### GV1001 alters microglia profiles and promotes transition into DAM2 population in 5xFAD mice

**Fig. 3:**
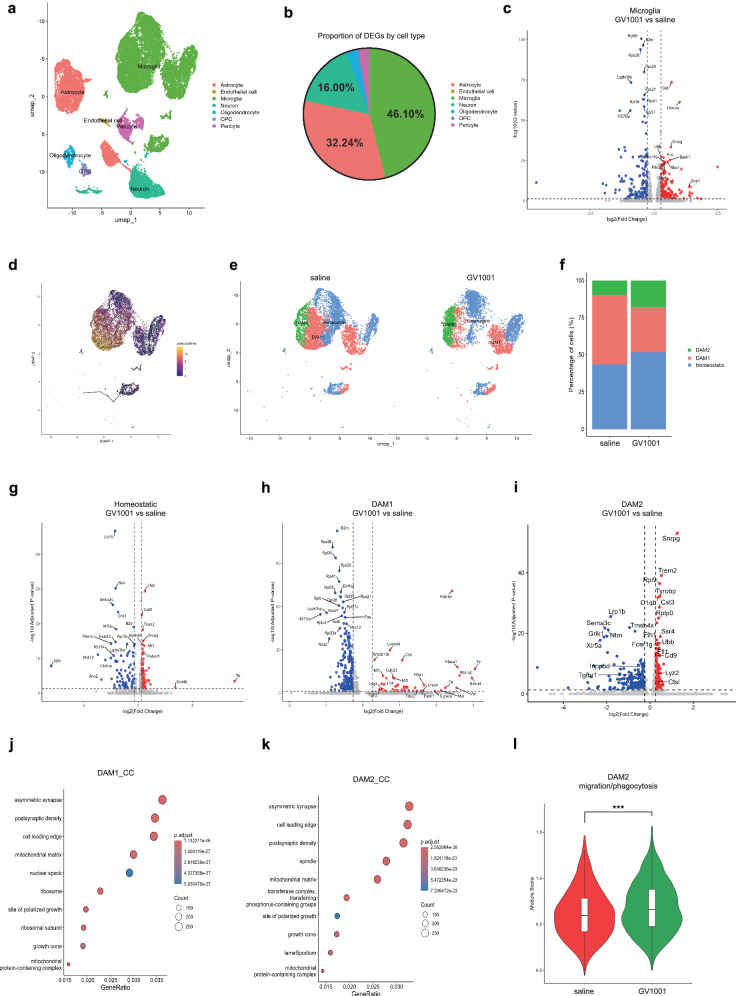
scRNA-seq reveals that GV1001 alters the disease-associated microglia profile. **a** Uniform Manifold Approximation and Projection (UMAP) of identified brain cell types in 5xFAD mice after GV1001 injection for 2 months (*n* = 4–6 mice per each group). **b** Proportions of DEGs across cell types. **c** Volcano plot of DEGs in all microglia between GV1001-injected and saline-injected 5xFAD mice (*q*-value < 0.05). **d**, **e** Pseudotime analysis identifying homeostatic, DAM1, and DAM2 microglial population. **f** Relative proportions of homeostatic, DAM1, and DAM2 microglia. **g–i** Volcano plots of DEGs in homeostatic, DAM1, and DAM2 microglia between GV1001-injected and saline-injected 5xFAD mice (*q*-value < 0.05). **j**,**k** CC analysis in DAM1 and DAM2 microglia (*P* < 0.05, false discovery rate corrected using Benjamini–Hochberg method). **l** Gene module enrichment related to migration and phagocytosis. CC, cellular component; DAM, disease-associated microglia; DEG, differentially expressed gene, OPC Oligodendrocyte Precursor Cell.

To gain better understanding of the effects of GV1001 on microglial modulation, we performed scRNA-seq on cortical cells isolated from 5xFAD mice injected with either saline or GV1001. Single-cell transcriptomic profiles of 24,319 cells (12,462 from saline and 11,857 from GV1001) were selected for analysis. Unsupervised clustering was performed using Seurat package, and the cells were projected onto a Uniform Manifold Approximation and Projection for visualization. We identified seven major cell types, including neurons, astrocytes, microglia, oligodendrocytes, oligodendrocyte progenitor cells, endothelial cells, and pericytes (Fig. 3a). Consistent with the previous scRNA-seq studies in 5xFAD mice, we obtained more microglia than other cell types^33,34^.

Next, we evaluated differentially expressed genes (DEGs) within each cell cluster and found that GV1001 injection in 5xFAD mice induced the most significant transcriptional changes in microglia compared with other brain cell types (Fig. 3b, c and Supplementary Fig. 6). The second largest transcriptional changes were observed in astrocytes. Gene ontology enrichment analysis revealed that astrocyte DEGs were primarily associated with pathways related to cytoplasmic translation, ribosomal assembly, mitochondrial oxidative phosphorylation, and protein quality control (Supplementary Fig. 6). Compared with these predominantly metabolic and proteostatic responses in astrocytes, microglia exhibit remarkable phenotypic heterogeneity, reflecting different activation states. To investigate whether GV1001 directly affects microglial heterogeneity and influences their transition between distinct states, we performed pseudotime analysis and identified homeostatic and disease-associated microglia (DAM) populations^14^. DAM is a unique subset of microglia spatially associated with sites of AD pathology and can be further classified into stage I DAM (DAM1), and stage II DAM (DAM2) subpopulations. DAM1 represents an intermediate stage, whereas DAM2 reflects a fully activated state characterized by enhanced phagocytic activity and lipid metabolism in AD (Fig. 3d and Supplementary Fig. 7).

Comparison of cell-type distributions between 5xFAD-saline and 5xFAD-GV1001 groups revealed that GV1001 significantly increased the proportion of homeostatic and DAM2 microglia (Fig. 3e, f). Additionally, GV1001 upregulated genes associated with the DAM transition, including *Trem2*, *Tyrobp*, *Fth1*, *Ftl1*, *Cd9*, *Lyz2*, and *Ctsl,* while downregulating homeostatic microglia genes such as *Tgfbr1* and the checkpoint *Inpp5d* in DAM2 microglia^14,35,36^ (Fig. 3i). By contrast, homeostatic and DAM1 microglia exhibited more complex changes in gene expression. GV1001 led to a decrease in several DAM and interferon-related genes (*Lgals3bp*, *B2m*, *Rpl27*, and *Rpl29*), yet also induced an increase in other key genes such as *Trem2* and *Lag3*, which are implicated in DAM function and interferon responses^37,38^ (Fig. 3g, h). Notably, despite an overall downregulation in inflammatory responses in the brain (Supplementary Fig. 2) and a concurrent increase in the homeostatic microglia portion, GV1001 also elevated the proportion of DAM2 microglia (Fig. 3e, f). Given that TREM2-dependent DAM2 formation is crucial for enhancing microglial phagocytic activity and lipid metabolism in response to Aβ plaques^14^, these findings suggest that GV1001 promotes the transition to DAM2, likely contributing to the reduction of Aβ plaque burden in the brain. Therefore, the observed gene expression changes in homeostatic and DAM1 microglia may represent secondary effects resulting from the increased DAM2 formation, leading to amyloid plaques clearance and a subsequent attenuation of overall immune responses.

To further investigate microglial changes, we analyzed cellular component (CC) in DAM1 and DAM2 populations using microglial DEGs (false discovery rate < 0.05, fold change > 1.2). Notably, cell migration-related genes — such as those involved in leading edge formation, polarized growth, growth cone development, and lamellipodium — were highly enriched (Fig. 3j, k), consistent with the observed increase in microglial migration around the amyloid plaques (Fig. 2f–j).

Additionally, gene module enrichment analysis revealed that migration-related and phagocytosis-related gene modules were upregulated following GV1001 treatment in DAM2 subpopulation (Fig. 3l). Microglial migration and phagocytosis of Aβ are essential for clearing Aβ plaques from the brain parenchyma^39,40^. Furthermore, exposure of microglia to Aβ plaques or apoptotic neurons serves as a key trigger for microglial phenotypic transition^41^. Previous studies have shown that microglial migration toward Aβ is impaired with aging, and that restoring this process enhances microglial phagocytic activity in APP/PS1 mice^39^. In sum, our single-cell transcriptomic analyses suggest that GV1001 promotes microglial migration and Aβ responsiveness by driving the transition toward the DAM2 state.

### GV1001 promotes microglia migration with lamellipodium formation

**Fig. 4:**
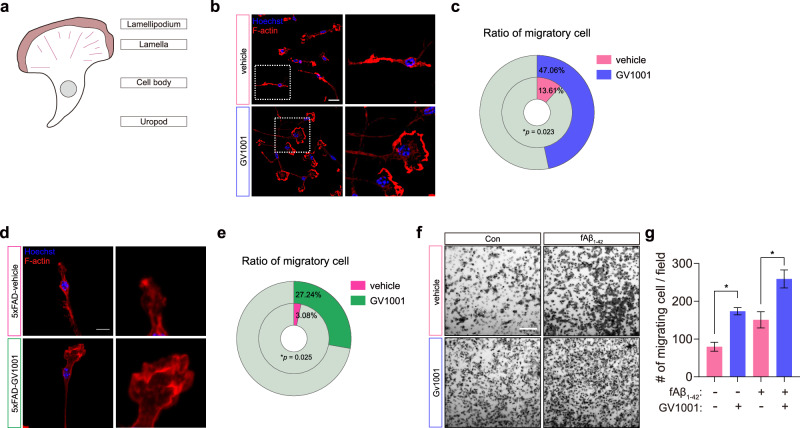
GV1001 promotes lamellipodium formation and migration of microglia. **a** Diagram of lamellipodium compartment in a cell. **b** Representative immunocytochemistry images of F-actin staining in primary cultured microglia treated with GV1001 (1 μM) or PBS (vehicle) for 6 h. Scale bar, 20 μm. **c** Quantification of the migratory microglia with F-actin accumulation in lamellipodium (*n* = 120–132 cells from three experiments). **d** Representative immunocytochemical images of F-actin staining in adult microglia acutely isolated from 8–9-month-old 5xFAD mice and treated with GV1001 (1 μM) or PBS (vehicle) for 6 h. Scale bar, 10 μm. **e** Quantifications of the migratory microglia with F-actin accumulation in lamellipodium (*n* = 110–128 cells from four experiments). **f** Transwell assay using primary cultured microglia treated with and without fAβ_1-42_ and GV1001. Scale bar, 20 μm. **g** Quantification of microglia migration (*n* = 3). All statistical comparisons were conducted with unpaired *t* test, *P < 0.05. Data are means ± SEM. Aβ, amyloid β.

On the basis of our transcriptomics results, we hypothesized that enhanced migration of microglia toward Aβ plaques may underlie GV1001-induced increase in the number of microglia around the Aβ plaques. During cell migration, filamentous actin (F-actin) assembly drives the formation of a membrane protrusion called lamellipodium, which is a separate region of the plasma membrane in the direction of cell movement (Fig. 4a), and the leading edge of the lamellipodium provides cell adhesion to the substratum and contraction for translocation^42,43^.

To examine whether GV1001 promotes lamellipodium formation in microglia for cell migration, we treated primary cultured microglia with GV1001 and observed F-actin accumulation by phalloidin staining as a surrogate marker of cell migration. GV1001 treatment efficiently induced accumulation of F-actin and lamellipodium formation with polarity, suggesting enhanced migratory behavior of microglia (Fig. 4b, c). Next, we acutely isolated adult microglia from 8–9-month-old 5xFAD mice and treated these cells with GV1001. Consistent with the results in primary microglia, GV1001 also promoted F-actin accumulation and lamellipodium formation with polarity in adult 5xFAD microglia (Fig. 4d, e). To confirm that an increase in F-actin accumulation leads to mobilization of microglia toward Aβ, we performed transwell cell migration assay. Aβ plaques have chemotactic effects on microglia^44^. As such, the fibrillar form of Aβ (fAβ_1-42_) increased microglia migration (Fig. 4f). GV1001 treatment significantly further increased microglia migration both in basal and in Aβ-treated conditions (Fig. 4f, g).

### Ligand-based virtual target screening reveals that GV1001 binds to bradykinin receptor 1

**Fig. 5:**
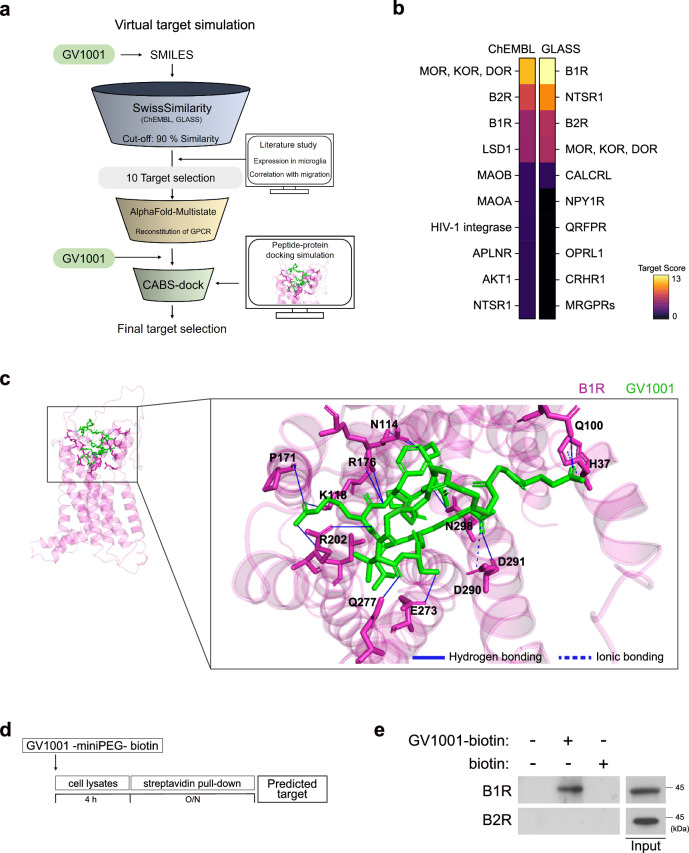
Virtual target screening and docking simulation suggest the binding of GV1001 to bradykinin receptor 1. **a** Diagram of virtual target screening and docking prediction. **b** Selection of 10 targets with high target scores from ligand-based virtual target screening (LBVS) using SwissSimilarity with ChEMBL and GLASS databases. **c** Visualization of protein residues predicted to interact with GV1001 peptide using the PyMOL. GV1001 and B1R are shown in green and pink, respectively. **d** Schematic diagram of biotin–streptavidin pulldown assay. **e** Representative western blots of B1R and B2R after streptavidin pulldown in GV1001-biotin-treated primary microglia culture. B1R, bradykinin receptor 1; B2R, bradykinin receptor 2; GPCR, G protein-coupled receptor.

To identify the signaling pathway responsible for migration and increased phagocytic activity induced by GV1001, we conducted virtual target screening and peptide docking simulation. SwissSimilarity and CABS-dock were used for ligand-based virtual target screening (LBVS)^45^ and peptide–protein docking prediction, respectively^26^ (Fig. 5a). For LBVS, we initially chose ChEMBL database^21,22^, which is a manually curated database of bioactive molecules with drug-like properties and found that GV1001 had high probability to bind to B1R, bradykinin receptor 2 (B2R), opioid receptors (OR, mu; MOR, kappa; KOR, delta; DOR), and lysine specific demethylase 1 (LSD1) (Fig. 5b). As most of targets highly ranked in ChEMBL through LBVS were GPCRs, we also performed screening with GPCR-ligand association (GLASS) database^23^, which is a curated repository for experimentally validated GPCR–ligand interaction. GV1001 again was predicted to bind to B1R, neurotensin receptor 1 (NTSR1), B2R, and ORs in GLASS database (Fig. 5b). To corroborate results of LBVS and narrow down the potential binding candidates of GV1001, peptide–protein docking simulation using CABS-dock was performed. We found that GV1001 is expected to fit into the binding pocket of B1R but no other possible targets. Interaction residues between B1R and GV1001 including hydrophobic interactions, hydrogen bonds, and ionic bonds analyzed from docking structure were visualized and listed (Fig. 5c and Supplementary Table 1). Within the 5 Å region of the binding site, GV1001 forms 14 hydrogen bonds and 18 hydrophobic interactions with B1R, suggesting possible strong binding affinity between GV1001 and B1R. Next, to assess whether GV1001 physically binds to B1R, we generated biotin-labeled GV1001. Biotin-labeled GV1001 demonstrated the same efficacy as un-labeled GV1001 for promotion of lamellipodium formation and phagocytic ability of microglia (Supplementary Fig. 8a,b). A biotin–streptavidin pulldown assay was performed with lysates of primary cultured microglia treated with GV1001-biotin (Fig. 5d) and confirmed the interaction of GV1001 with B1R but not with B2R (Fig. 5e). In sum, these data suggest that B1R is a genuine target of GV1001.

### B1R is required for the rescue of neurodegeneration by GV1001 in 5xFAD mice

Bradykinin receptors B1R and B2R are the targets of bradykinin (BK) peptides produced by the action of kallikrein enzymes from kininogens^46^. Kallikrein-kinin system is a complex peptide hormonal system involved in various physiological processes including vasodilation, inflammation, pain control, and thermogenesis^46^. The main components of kallikrein-kinin system are BK, Lys-BK with high affinity to B2R, whereas B1R preferentially binds to C-terminal des-Arg metabolites of BK or Lys-BK^47,48^.

**Fig. 6:**
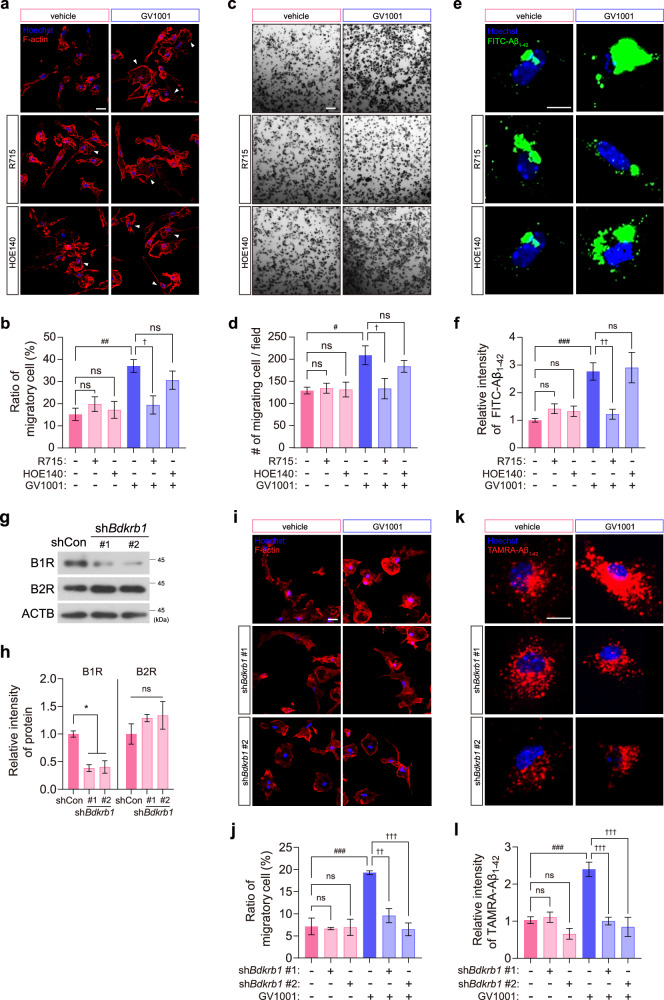
Inhibition of bradykinin receptor 1 blocks GV1001-induced microglia migration and phagocytosis. **a** Representative immunocytochemical images of F-actin staining in primary cultured microglia treated with GV1001 (1 μM) and inhibitors against B1R (R715; 1 μM) or B2R (HOE140; 1 μM). Scale bar, 10 μm. **b** Quantification of F-actin accumulation in lamellipodium (*n* = 80–101 cells from four experiments). **c** Transwell assay using primary cultured microglia treated with GV1001 and R715 or HOE140. Scale bar, 10 μm. **d** Quantification of microglia migration (*n* = 5). **e** Representative fluorescence images of fluorescein isothiocyanate (FITC)-fAβ_1-42_ uptake by primary cultured microglia with GV1001 (1 μM) and R715 or HOE140. Scale bar, 10 μm. **f** Quantification of FITC-fAβ_1-42_ intensity in microglia (*n* = 94–112 cells from four experiments). **g** Representative western blots of B1R and B2R in microglia transduced with lentivirus expressing short hairpin RNA targeting two different sequences of *Bdkrb1* (encoding B1R). **h** Mean protein levels after normalization of ACTB (*n* = 4). **i** Representative immunocytochemical images of F-actin staining in microglia transduced with lentivirus expressing shCon or sh*Bdkrb1* with or without GV1001. Scale bar, 10 μm. **j** Quantification of F-actin accumulation in lamellipodium (*n* = 135–169 cells from three experiments). **k** Representative fluorescence images of TAMRA-fAβ_1__–42_ uptake by microglia transduced with lentivirus expressing shCon or sh*Bdkrb1* with or without GV1001. Scale bar, 10 μm. **l** Quantification of TAMRA-fAβ_1__–42_ intensity in microglia (*n* = 88–152 cells from four experiments). All statistical comparisons were conducted with one-way analysis of variance followed by a post hoc test (parametric and non-parametric) as per applicable, *P < 0.05, **P < 0.01, and ***P < 0.001; ns, not significant. Data are means ± SEM. B1R, bradykinin receptor 1; B2R, bradykinin receptor 2; shCon, non-targeting control shRNA.

To examine whether the effects of GV1001 are dependent on B1R, we co-treated microglia with GV1001 and R715 or HOE140, which are the B1R-specific and B2R-specific inhibitors, respectively. Consistent with our in silico prediction with B1R as the top candidate by both LBVS and docking simulation, R715 but not HOE140 blocked GV1001-induced migration (Fig. 6a−d) and phagocytosis (Fig. 6e, f and Supplementary Fig. 9) of microglia. As ORs were also suggested as possible targets of GV1001 by LBVS but not in docking simulation (Fig. 5b), we chose DOR to further validate our in silico prediction and found that naltrindole, the specific inhibitor of DOR, failed to block the effects of GV1001 on F-actin formation and migration of microglia (Supplementary Fig. 10a−d), confirming that B1R may be the specific target molecule which mediates the therapeutic effects of GV1001. To corroborate our results obtained with the pharmacological inhibitors, we genetically knocked down the *Bdkrb1* (encoding B1R) gene in primary cultured microglia by transduction with lentivirus expressing two different *Bdkrb1*-targeting short hairpin RNAs (Fig. 6g, h). In a similar manner to the pharmacological inhibition, genetic suppression of B1R expression abrogated the effects of GV1001 on migration (Fig. 6i, j) and phagocytosis of microglia (Fig. 6k, l).

**Fig. 7:**
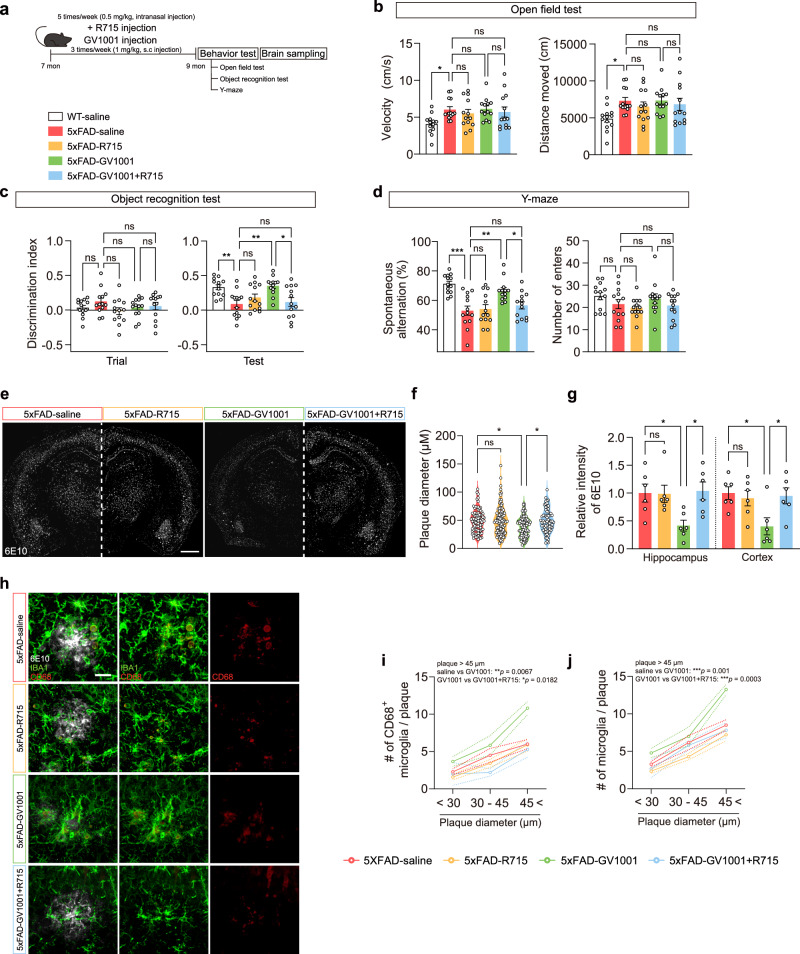
Inhibition of bradykinin receptor 1 blocks memory recovery and amyloidolytic effects of GV1001 in 5xFAD mice. **a** Diagram of the experimental schedule. Open-field test (part **b**) for locomotion analysis, object recognition test (part **c**) and Y-maze test (part **d**) for memory assessment in wild-type (WT) and 5xFAD mice (*n* = 13 mice per group) after R715 and GV1001 injection. **e** Representative immunofluorescence images obtained by staining with 6E10 antibody in brains of 8–9-month-old 5xFAD mice injected with R715 and GV1001. Scale bar, 1 mm. Quantification of plaque size (part **f**) and 6E10 intensity (part **g**) in the hippocampus (left) and cortex (*n* = 6 mice per group). **h** Representative immunofluorescence images obtained by co-staining with 6E10 (Aβ plaques), IBA1 (microglia), and CD68 (phagocytic microglia) antibodies in the hippocampus of 8–9-month-old 5xFAD mice (*n* = 5 mice per group) injected with R715 and GV1001. Scale bar, 20 μm. Analysis of the number of CD68^+^ phagocytic microglia (part **i**) and total microglia (part **j**) per plaque depending on the size of Aβ plaques (*n* = 5 mice per group). All statistical comparisons were conducted with one-way analysis of variance followed by Tukey’s multiple comparisons test, *P < 0.05, **P < 0.01, and ***P < 0.001; ns, not significant. Data are means ± SEM. s.c., subcutaneous.

To investigate whether R715 blocks the effects of GV1001 in 5xFAD mice, we intranasally injected R715 as previously reported^49,50^ (Fig. 7a). Hyperlocomotion activity of 5xFAD mice in OFT test was not affected by R715 administration (Fig. 7b). In memory tests, R715 did not exacerbate the memory loss of 5xFAD (Fig. 7c,d). However, of note, R715 significantly prevented the memory recovery in GV1001-injected 5xFAD mice both in object recognition test (Fig. 7c) and Y-maze assay (Fig. 7d), supporting that memory rescue effect of GV1001 is dependent on B1R.

Next, we wondered whether R715 can block the Aβ plaques reduction and recruitment of microglia induced by GV1001 administration in 5xFAD mice. We observed B1R expression in plaque-associated microglia (Supplementary Fig. 11a,b). Co-injection of R715 with GV1001 abolished the reduction of Aβ plaques in both the hippocampus and cortex of 5xFAD mice (Fig. 7e−g). In addition, blockade of B1R prevented recruitment of both CD68^+^ and total microglia induced by GV1001 in association with large plaques (diameter > 45 μm; Fig. 7h–j). Taken together, B1R inhibition blocked the neuroprotective effects of GV1001 in cognitive improvement and alleviation of amyloid burden by preventing GV1001-inducedmicroglial migration and Aβ degradation toward large Aβ plaques.

### mTORC2 regulates GV1001-induced microglia phagocytosis and migration with lamellipodium formation

**Fig. 8:**
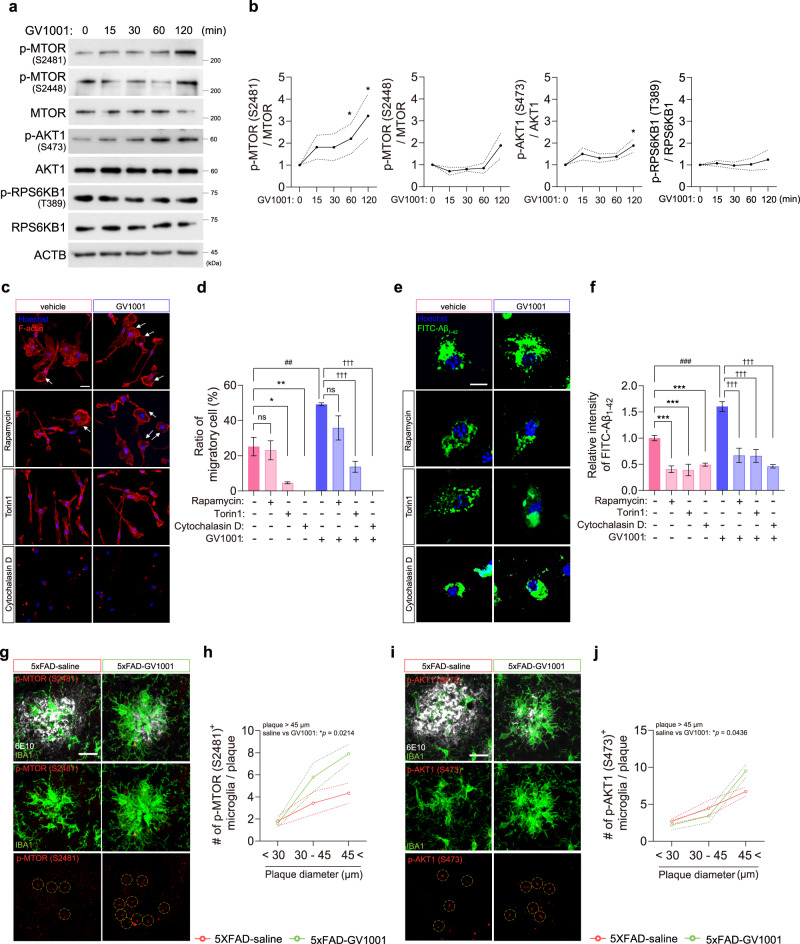
mTORC2 mediates GV1001-induced microglia phagocytosis and migration with lamellipodium formation. **a** Representative western blots showing the time course of mTORC1 and mTORC2 activation in primary cultured microglia after GV1001 treatment. **b** Mean levels of phosphorylated (p) mTOR at S2448 and S2481, AKT1 at S473, and RPS6KB1 at T389 after normalization to the total form of each protein (*n* = 4). **c** Representative immunocytochemical images of F-actin staining in GV1001-treated primary cultured microglia after treatment with inhibitors against mTORC1 (rapamycin; 400 nM), mTORC2 (Torin1; 500 nM), and actin polymerization (cytochalasin D; 10 μM). Scale bar, 10 μm. **d** Quantification of F-actin accumulation in lamellipodium (*n* = 53–67 cells from three experiments). **e** Representative fluorescence images of fluorescein isothiocyanate (FITC)-fAβ_1-42_ uptake by primary cultured microglia with rapamycin, Torin1, and cytochalasin D. Scale bar, 10 μm. **f** Quantification of FITC-fAβ_1-42_ intensity in microglia (*n* = 54–145 cells from three experiments). **g** Representative immunofluorescence images obtained by co-staining with 6E10 (Aβ plaques), IBA1 (microglia), and p-mTOR (S2481; mTORC2) antibodies of the hippocampus of 8–9-month-old 5xFAD mice. Scale bar, 10 μm. **h** Analysis of the number of p-mTOR S2481^+^ microglia per plaque depending on the size of Aβ plaques (*n* = 6 mice per group). **i** Representative immunofluorescence images obtained by co-staining with 6E10 (Aβ plaques), IBA1 (microglia), and p-AKT1 (S473) antibodies of the hippocampus of 8–9-month-old 5xFAD mice. Scale bar, 10 μm. **j** Analysis of the number of p-AKT1 S473^+^ microglia per plaque depending on the size of Aβ plaques (*n* = 6 mice per group). Unpaired *t* test (parts **h** and **j**) and one-way analysis of variance followed by Tukey’s multiple comparisons test (parts **b**, **d** and **f**) were conducted, *P < 0.05, **P < 0.01, and ***P < 0.001; ns, not significant. Data are means ± SEM.

Our data showed that GV1001 promotes migration and phagocytosis of microglia through B1R both in vitro and in vivo. To reveal signaling pathway downstream of B1R activation which is a crucial player for cell migration^51,52^, we focused on mTOR as downstream signaling molecule of B1R^53–55^. mTOR is a serine/threonine kinase with two distinct forms: mTOR complex 1 (mTORC1) and 2 (mTORC2)^56^. mTOR is involved in various cellular events, including determination of cell size, proliferation, survival, and autophagy^57,58^. Another important mTOR-dependent cellular process is cytoskeleton dynamics and organization^59,60^. Thus, mTORC2 activity declines with age in the brain of both fruit flies and rodents and loss of mTORC2-mediated actin polymerization contributes to age-associated memory loss^61^. As GV1001 induced F-actin accumulation (Fig. 4b−e), we first examined whether GV1001 alters mTOR signaling in microglia. GV1001 induced rapid phosphorylation of mTOR S2481 from as early as 15 min, which was the earliest time point we observed and phosphorylation at S2481 continuously increased until 2 h, the latest time point we observed (Fig. 8a, b). However, phosphorylation of mTOR S2448 remained low and began to increase at the 2 h time point (Fig. 8a, b). Phosphorylation at S2448 and S2481 reflects activation of mTORC1 and mTORC2, respectively^56^. Therefore, our time course analysis suggests mTORC2 activation as the primary responder to GV1001. The well-known effectors of mTORC1 and mTORC2 are RPS6KB1 (also known as p70S6K) and AKT1, respectively^56^. Consistently, GV1001 treatment significantly increased phosphorylation of AKT1 at S473, whereas RPS6KB1 phosphorylation at T389 was not altered until 2 h (Fig. 8a, b). Overall, these data suggest that GV1001 activates mTORC2 rather than mTORC1.

mTOR is recruited in membrane leading edge of migrating cells by GPCR^62^. We found that migratory cells showed p-mTOR (S2481) accumulation in the leading edge of microglia (Supplementary Fig. 12a−c) and GV1001 treatment increased p-mTOR (S2481)^+^ lamellipodium in microglia (Supplementary Fig. 12d,e), supporting the engagement of mTORC2 in GV1001-induced microglia migration. To examine whether GV1001-induced mTORC2 activation promotes migration and phagocytosis of microglia, we used rapamycin and Torin 1, inhibitors of mTORC1 and mTORC1/2, respectively^63^. mTORC1 includes a scaffolding protein, RAPTOR, which is sensitive to the immunosuppressant rapamycin, whereas mTORC2 contains RICTOR, which is relatively resistant to short-term treatment with rapamycin^64,65^. However, Torin 1 can inhibit both mTORC1 and mTORC2 (ref. ^63^). Rapamycin treatment did not alter lamellipodium formation in microglia either in the basal state or with GV1001 treatment (Fig. 8c, d). By contrast, Torin 1 significantly decreased lamellipodium formation and changed polarized cell morphology to the non-migratory shape, indicating that actin cytoskeleton dynamics in microglia is regulated by mTORC2 and GV1001 acts primarily through the mTORC2 pathway (Fig. 8c, d). The concentrations of rapamycin and Torin 1 used here were verified in our previous study in primary cultured microglia^9^. Therefore, we compared the inhibitory effect of Torin 1 on actin reorganization with those of cytochalasin D, which inhibits actin polymerization. As expected, both inhibitors effectively blocked F-actin formation and eliminated cell polarity (Fig. 8c, d). Taken together, these data suggest that mTORC2 is the key mediator of the effects of GV1001 on actin cytoskeleton reorganization for migration.

Our data showed that GV1001 not only promoted microglia clustering around Aβ in 5xFAD mice but also increased the number of CD68^+^ phagocytic microglia near the plaques (Fig. 2f). Also, we observed that GV1001 treatment significantly increased microglial fAβ_1-42_ uptake (Fig. 6e). Of note, Torin 1 blocked fAβ_1-42_ uptake at basal state and abolished the effects of GV1001 on Aβ engulfment (Fig. 8e, f). Unlike the absence of an effect on lamellipodium formation, rapamycin also prevented fAβ_1-42_ uptake both at basal state and after GV1001 treatment (Fig. 8e, f), suggesting that activation of both mTORC1 and mTORC2 can enhance Aβ uptake by microglia. Moreover, we examined whether engulfed fAβ_1-42_ is degraded by the lysosomal pathway by performing proximity ligation assay between fAβ_1-42_ and LAMP2, a lysosome membrane protein (Supplementary Fig. 12f,g). GV1001 increased the proximity ligation assay signal, indicating that GV1001 indeed increases lysosomal fAβ_1-42_ clearance in microglia.

To verify that GV1001 activates mTORC2 in vivo, we monitored p-mTORC2 (S2481) and p-AKT1 (S473) by immunohistochemistry in 5xFAD mice (Fig. 8g−j). Immunoreactive signals of p-mTORC2 (S2481) and p-AKT1 (S473) were detected in microglia engaged with Aβ plaques, and GV1001 greatly increased their immunoreactivity, especially in large plaques, confirming that GV1001 indeed activates mTORC2 in vivo (Fig. 8g−j).

**Fig. 9:**
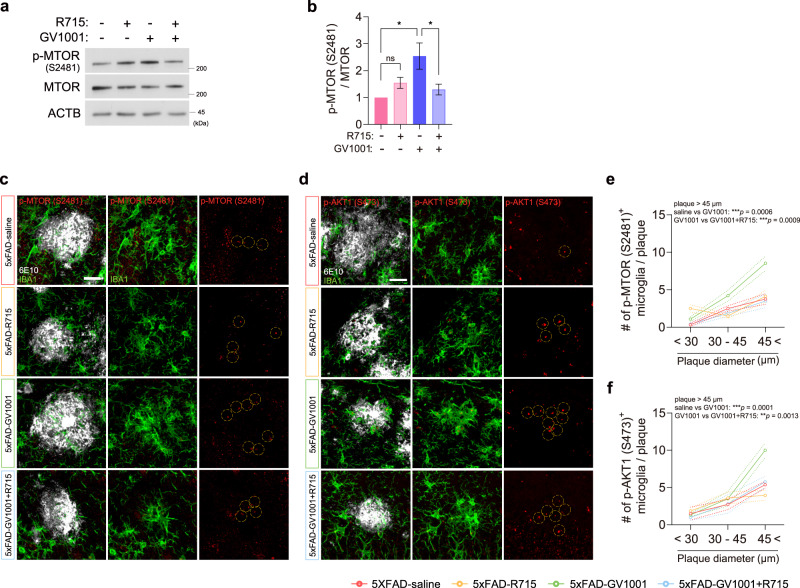
Inhibition of bradykinin receptor 1 blocks MORTC2 activation by GV1001 in microglia. **a** Representative western blots showing the activation of mTORC2 in primary cultured microglia after GV1001 and R715 treatment. **b** Mean levels of phosphorylated (p) mTOR at S2481 after normalization to the total form of mTOR (*n* = 4). **c** Representative immunofluorescence images obtained by co-staining with 6E10 (Aβ plaques), IBA1 (microglia), and p-mTOR (S2481; mTORC2) antibodies of the hippocampus of 8–9-month-old 5xFAD mice. Scale bar, 10 μm. **d** Analysis of the number of p-mTOR S2481^+^ microglia per plaque depending on the size of Aβ plaques (*n* = 5 mice per group). **e** Representative immunofluorescence images obtained by co-staining with 6E10 (Aβ plaques), IBA1 (microglia), and p-AKT1 (S473) antibodies of the hippocampus of 8–9-month-old 5xFAD mice. Scale bar, 10 μm. **f** Analysis of the number of p-AKT1 S473^+^ microglia per plaque depending on the size of Aβ plaques (*n* = 5 mice per group). All statistical comparisons were conducted with one-way analysis of variance followed by Tukey’s multiple comparisons test, *P < 0.05, **P < 0.01, and ***P < 0.001; ns, not significant. Data are means ± SEM.

Next, we wondered whether mTORC2 activation is dependent on B1R. We treated primary microglia with GV1001 for 1 h, which showed significant increase in p-mTOR (S2481), but inhibition of B1R by R715 significantly blocked GV1001-triggered mTORC2 activation (Fig. 9a, b). To examine whether R715 blocks the mTORC2 activation by GV1001 in vivo, we monitored p-mTORC2 (S2481) and p-AKT1 (S473) in 5xFAD mice after injection of GV1001 with R715. Increased immunoreactivity of p-mTORC2 (S2481) and p-AKT1 (S473) by GV1001 injection was abolished by R715 co-administration. However, injection of R715 alone did not alter mTORC2-AKT1 signaling of microglia at basal state (Fig. 9c−f).

To verify that the increase in the DAM2 proportion induced by GV1001 is B1R-dependent, we stained DAM2 microglia by using CLEC7A. CLEC7A is a pattern recognition receptor which is expressed in microglia. CLEC7A^+^ microglia are located in neurogenic area and increased by aging^66^. In AD, CLEC7A is highly upregulated in DAM2 and CLEC7A-induced activation of SYK alters AKT1 signal and increases clearance of Aβ^67^. Therefore, we examined whether GV1001 increases CLEC7A^+^ microglia near the Aβ plaque. Consistent with scRNA-seq data, immunohistochemical analyses revealed that GV1001 increased CLEC7A^+^ DAM2 microglia. Furthermore, R715 significantly prevented the GV1001-induced increase in DAM2 microglia associated with large Aβ plaques (Supplementary Fig. 13). These findings suggest that GV1001 promotes DAM2 formation, and that this phenotypic shift is at least partially dependent on B1R.

Collectively, our in vitro and in vivo analyses suggest that GV1001 induces microglial migration through actin cytoskeleton reorganization and enhances DAM2 formation, thereby promoting Aβ clearance. Additionally, B1R-mediated mTORC2-AKT1 pathway appears to be, at least in part, a critical mediator of GV1001’s therapeutic effects in 5xFAD mice.

## Discussion

Current clinically available options for patients with AD, including cholinesterase and glutamate receptor inhibitors, provide only symptomatic benefits, and identification of disease-modifying drugs remains a big challenge. Numerous drugs focused on Aβ production or its direct neutralization and clearance have failed to reach clinical end points, except aducanumab, which was approved by the FDA through the accelerated pathway in 2021 (ref. ^68^). However, owing to the high rates of serious side effects as well as uncertain clinical benefits and high cost of mediation, aducanumab was withdrawn from the market. Recently, lecanemab was also approved by the FDA^69^ and donanemab is waiting for approval^70^. Both significantly slowed down cognitive decline in patients with early AD. However, lecanemab caused brain swelling and bleeding, especially in patients with a condition of cerebral amyloid angiopathy^71^. In addition, donanemab appears to come with higher risks than lecanemab^72^. Since they target the early stage of patients with AD, it awaits further evaluation on whether they are truly disease-modifying agents. Besides, various anti-inflammatory drugs have also failed to yield substantial benefits in patients with AD. Thus, there is huge unmet medical need for the prevention and treatment of AD with proven safety.

Recent studies have highlighted the importance of microglial modulation in AD pathogenesis. However, no available drugs have been developed to achieve this yet. Of note, a recent phase II clinical trial with GV1001 showed promising efficacy with cognitive improvement in patients with moderate-to-severe AD without safety concerns^20^. However, whether GV1001 can modulate microglia profile remained unclear. Here, we show that GV1001 effectively modified the pathogenesis in 5xFAD mice, leading to a profound improvement in amyloid pathology and recovery of synaptic integrity and memory. GV1001 induced mobilization of microglia but not astrocytes toward Aβ deposition. In particular, GV1001-induced increase in microglial engagement with Aβ plaques was accompanied by an increase in the number of phagocytic microglia around large plaques. Consistently, our in vitro and scRNA-seq analyses demonstrated that GV1001 promotes microglial migration by inducing actin cytoskeleton reorganization and lamellipodium formation, thereby facilitating Aβ engulfment by microglia.

In our search for the binding target and relevant cellular signaling mechanisms underlying the therapeutic effects of GV1001, we unveiled the key participation of B1R–mTORC2 signaling axis. Virtual target screening and peptide docking simulation predicted B1R as the potential binding target of GV1001. This in silico prediction was validated by biochemical peptide pulldown assay and pharmacological and genetic inactivation of B1R. As the effects of GV1001 on F-actin formation and Aβ uptake were fast within several hours and remained robust (Figs. 4 and 8), we explored the possibility of a rapidly responding signaling mechanism rather than transcriptional regulation and identified mTORC2 as a critical downstream transducer of B1R activation. Once activated by GV1001, B1R–mTORC2 axis drove both lamellipodium formation for migration and Aβ clearance (Fig. 8), although mTORC1 also contributed to Aβ engulfment.

BK and its receptors have an important role in neurodegeneration, especially in the pathogenesis of AD^73^. Pharmacological or genetic inactivation of B1R and B2R reversed Aβ injection-induced cognitive decline in rats^74^. Another study also reported that blockade of B1R improved cognitive function^75^, suggesting the pathogenic role of B1R in AD. However, growing evidence also supports the neuroprotective role of B1R in AD. Although BK and its receptor are considered pro-inflammatory in the periphery, BRs possess anti-inflammatory and neuroprotective effects especially in glial cells^76,77^. Also, microglia recruitment around the lesion is dependent on B1R, supporting the view that B1R may have distinct roles in the brain especially in microglia^78^. In line with this view, administration of R715 in early months of 5xFAD or Tg-SwDI mice reduced the recruitment of microglia around the plaque and increased the size of amyloid plaques^49,78^. Of note, the human B1R level was reduced in the fusiform gyrus of patients with AD, indicating that B1R signaling may be downregulated in patients with AD^79,80^.

Importantly, differences in pharmacological tools used to interrogate B1R function may contribute to divergent conclusions across studies. The study reporting improved cognitive outcomes following B1R blockade used SSR240612 (ref. ^75^), a non-peptide antagonist with high affinity for B1R but incomplete receptor selectivity (Ki 0.48–0.73 nM for human B1R versus 358–481 nM for B2R)^81^. Although SSR240612 does not functionally antagonize B2R-mediated signaling at submicromolar concentrations, given the continuous high-dose minipump administration used in that study (10 mg/kg/day in 40% dimethyl sulfoxide), partial engagement of B2R cannot be fully excluded. By contrast, our study used R715, a peptide antagonist that is widely characterized as highly selective for B1R, with potent low-nanomolar functional antagonism and no detectable activity at B2R^82,83^. We therefore propose that differences in antagonist specificity and pharmacological behavior between SSR240612 and R715 may underlie the apparent discrepancies between prior report and our findings.

Although R715 blocked the beneficial effects of GV1001, we did not observe significant increase in plaque load in 5xFAD mice injected with R715 alone, unlike the previous reports^49,78^. We assume that these differences stemmed from the different ages of mice used in the study. We injected R715 in 6–7 months of 5xFAD, which show clear amyloid deposition whereas previous studies were conducted at earlier months before robust Aβ deposition begins in 5xFAD or Tg-SwDI mice^49,78^.

GV1001 was reported to protect primary cultured neural stem cells from oxidative stress and Aβ toxicity. In these studies, GV1001 showed anti-apoptotic, anti-oxidant effects and stabilized mitochondria, leading to the recovery of neural stem cell viability and function^84,85^. These results suggest that GV1001 not only modulates microglia function but also possesses other modes of neuroprotection. Numerous clinical trials testify that drugs which affect single molecular target have failed in the treatment of AD^86,87^, as AD is characterized with increased apoptosis, oxidative stress, mitochondrial instability, and decreased neurogenesis as well as amyloid deposition, neurofibrillary tangle formation, and neuroinflammation^88^. Therefore, targeting multi-aspect of AD is desirable and, in that aspect GV1001 might be a promising therapeutic agent for AD. As B1R is expressed in other neural cells including neurons, it will be interesting to examine whether neuroprotective functions of GV1001 in other cell types are also mediated by B1R activation.

Microglia have heterogeneous activation status consistent with their diverse roles. In this study, we comprehensively examined microglial profile changes induced by GV1001 using scRNA-seq. We found that GV1001 increased the proportion of DAM2 microglia, suggesting that this peptide modulates microglial state transitions. To further support these findings, we analyzed CLEC7A⁺ microglia, which are representative of the DAM2 population and have a key role in Aβ clearance. Notably, we identified that the GV1001-induced increase in CLEC7A⁺ microglia was regulated by B1R. Collectively, our analyses demonstrate that GV1001 promotes microglial transitions in the context of AD and that DAM2 formation induced by GV1001 is, at least in part, B1R-dependent.

Beyond its primary effects on microglia, our scRNA-seq data also revealed that GV1001 significantly impacts astrocytes. Although it remains unclear whether these astrocytic changes are mediated indirectly through microglia or directly via astrocytic B1R signaling, the observed alterations in genes such as *Lgals3bp* and *Inpp5d* — identified through microglial DEG analysis — support the notion of active microglia–astrocyte crosstalk. Both *Lgals3bp* and *Inpp5d* have been reported as crucial regulators of astrocytes activity through microglial signaling^36,38^. In Supplementary Fig. 6, the transcriptional changes observed in astrocytes suggest a shift toward a reactive yet non-inflammatory and non-proliferative astrocytic state. GV1001-treated astrocytes showed increased expression of mitochondrial genes (*mt-Cytb*, *mt-Atp6*, and *mt-Co2*) and ribosomal components (*RPL/RPS* family), reflecting metabolic remodeling and enhanced translational capacity — hallmarks of astrocyte activation in neuroinflammatory contexts to meet elevated energetic demands^89^. Upregulation of molecular chaperones (*Hspa8* and *Hspa5*) further aligns with reactive astrocyte states in neurodegeneration^90^, highlighting enhanced protein quality control and cellular stress adaptation. Notably, these activation-associated features occurred alongside reduced expression of classical markers of inflammatory and proliferative astrogliosis such as *Gfap*, whereas *Apoe* expression was increased^38,91^. This dissociation suggests that GV1001 does not induce a canonical inflammatory or proliferative astrocytic phenotype, but instead promotes a responsive and supportive state. Mechanistically, this phenotype is consistent with concurrent microglial transcriptional changes induced by GV1001. Reduced microglial *Inpp5d* expression implies loosening of inhibitory checkpoints that normally constrain microglia–astrocyte communication, whereas downregulation of *Lgals3bp* — a microglia-derived factor known to promote astrocyte proliferation — likely limits excessive astrocytic proliferation and inflammatory reactivity^36,38,92^. Supporting this interpretation, decreased GFAP immunoreactivity in the cortex (Supplementary Fig. 2) confirmed reduced astrocytic reactivity. Collectively, these findings suggest a model in which GV1001 enhances microglia–astrocyte interaction while qualitatively reshaping its outcome, favoring metabolic and proteostatic activation of astrocytes without triggering overt inflammatory or proliferative astrogliosis. Such reprogramming of glial crosstalk may underlie the neuroprotective effects of GV1001 by stabilizing the tissue environment while preventing maladaptive glial overactivation. Future studies exploring this cell–cell interaction could provide valuable insights into the dynamic relationship between microglia and astrocytes in response to GV1001.

Although addressing these points requires further in-depth study, our findings will pave the groundwork for a molecular understanding of the beneficial effect of GV1001 on microglia. Furthermore, the mechanistic clues on the action of GV1001 presented in this work will contribute to a more rational design of study in the clinical setting and will accelerate more refinement of GV1001 as a novel AD therapeutic agent.

## Supplementary information

Supplementary Information

## References

[1] Selkoe, D. J. Preventing Alzheimer’s disease. *Science* **337**, 1488–1492 (2012). 22997326 10.1126/science.1228541

[2] Karran, E. & De Strooper, B. The amyloid hypothesis in Alzheimer disease: new insights from new therapeutics. *Nat Rev Drug Discov* **21**, 306–318 (2022). 35177833 10.1038/s41573-022-00391-w

[3] Heppner, F. L. et al. Immune attack: the role of inflammation in Alzheimer disease. *Nat Rev Neurosci* **16**, 358–372 (2015). 25991443 10.1038/nrn3880

[4] Tansey, K. E. et al. Genetic risk for Alzheimer’s disease is concentrated in specific macrophage and microglial transcriptional networks. *Genome Med* **10**, 1–10 (2018). 29482603 10.1186/s13073-018-0523-8 PMC5828245

[5] Novikova, G. et al. Integration of Alzheimer’s disease genetics and myeloid genomics identifies disease risk regulatory elements and genes. *Nat Commun* **12**, 1610 (2021). 33712570 10.1038/s41467-021-21823-y PMC7955030

[6] Wightman, D. P. et al. A genome-wide association study with 1,126,563 individuals identifies new risk loci for Alzheimer’s disease. *Nat Genet* **53**, 1276–1282 (2021). 34493870 10.1038/s41588-021-00921-z PMC10243600

[7] Song, W. M. & Colonna, M. The identity and function of microglia in neurodegeneration. *Nat Immunol* **19**, 1048–1058 (2018). 30250185 10.1038/s41590-018-0212-1

[8] Condello, C. et al. Microglia constitute a barrier that prevents neurotoxic protofibrillar Aβ42 hotspots around plaques. *Nat Commun* **6**, 6176 (2015). 25630253 10.1038/ncomms7176 PMC4311408

[9] Lee, J.-W. et al. TLR4 (Toll-like receptor 4) activation suppresses autophagy through inhibition of FOXO3 and impairs phagocytic capacity of microglia. *Autophagy* **15**, 753–770 (2019). 30523761 10.1080/15548627.2018.1556946 PMC6526818

[10] Nam, H. et al. Presenilin 2 N141I mutation induces hyperactive immune response through the epigenetic repression of REV-ERBα. *Nat Commun* **13**, 1972 (2022). 35418126 10.1038/s41467-022-29653-2 PMC9008044

[11] Ulland, T. K. et al. TREM2 maintains microglial metabolic fitness in Alzheimer’s disease. *Cell* **170**, 649–663.e13 (2017). 28802038 10.1016/j.cell.2017.07.023 PMC5573224

[12] Sierksma, A. et al. Novel Alzheimer risk genes determine the microglia response to amyloid-β but not to TAU pathology. *EMBO Mol Med* **12**, e10606 (2020). 31951107 10.15252/emmm.201910606 PMC7059012

[13] Deczkowska, A. et al. Disease-associated microglia: a universal immune sensor of neurodegeneration. *Cell* **173**, 1073–1081 (2018). 29775591 10.1016/j.cell.2018.05.003

[14] Keren-Shaul, H. et al. A unique microglia type associated with restricting development of Alzheimer’s disease. *Cell* **169**, 1276–1290.e17 (2017). 28602351 10.1016/j.cell.2017.05.018

[15] Shay, J. W. & Wright, W. E. Telomerase therapeutics for cancer: challenges and new directions. *Nat Rev Drug Discov* **5**, 577–584 (2006). 16773071 10.1038/nrd2081

[16] Greten, T. F. et al. A phase II open label trial evaluating safety and efficacy of a telomerase peptide vaccination in patients with advanced hepatocellular carcinoma. *BMC Cancer* **10**, 1–7 (2010). 20478057 10.1186/1471-2407-10-209 PMC2882353

[17] Bernhardt, S. et al. Telomerase peptide vaccination of patients with non-resectable pancreatic cancer: a dose escalating phase I/II study. *Br J Cancer* **95**, 1474–1482 (2006). 17060934 10.1038/sj.bjc.6603437 PMC2360729

[18] Brunsvig, P. F. et al. Telomerase peptide vaccination: a phase I/II study in patients with non-small cell lung cancer. *Cancer Immunol Immunother* **55**, 1553–1564 (2006). 16491401 10.1007/s00262-006-0145-7 PMC11030882

[19] Kyte, J. A. et al. Telomerase peptide vaccination combined with temozolomide: a clinical trial in stage IV melanoma patients telomerase vaccine and temozolomide in stage IV melanoma. *Clin Cancer Res* **17**, 4568–4580 (2011). 21586625 10.1158/1078-0432.CCR-11-0184

[20] Koh, S.-H. et al. Efficacy and safety of GV1001 in patients with moderate-to-severe Alzheimer’s disease already receiving donepezil: a phase 2 randomized, double-blind, placebo-controlled, multicenter clinical trial. *Alzheimers Res Ther* **13**, 1–11 (2021). 33771205 10.1186/s13195-021-00803-w PMC7995588

[21] Zdrazil, B. et al. The ChEMBL Database in 2023: a drug discovery platform spanning multiple bioactivity data types and time periods. *Nucleic Acids Res* gkad1004 **52**, D1180–D1192 (2023). 10.1093/nar/gkad1004 PMC10767899 37933841

[22] Davies, M. et al. ChEMBL web services: streamlining access to drug discovery data and utilities. *Nucleic Acids Res* **43**, W612–W620 (2015). 25883136 10.1093/nar/gkv352 PMC4489243

[23] Chan, W. K. et al. GLASS: a comprehensive database for experimentally validated GPCR–ligand associations. *Bioinformatics* **31**, 3035–3042 (2015). 25971743 10.1093/bioinformatics/btv302 PMC4668776

[24] Pándy-Szekeres, G. et al. GPCRdb in 2023: state-specific structure models using AlphaFold2 and new ligand resources. *Nucleic Acids Res* **51**, D395–D402 (2023). 36395823 10.1093/nar/gkac1013 PMC9825476

[25] Blaszczyk, M. et al. Modeling of protein–peptide interactions using the CABS-dock web server for binding site search and flexible docking. *Methods* **93**, 72–83 (2016). 26165956 10.1016/j.ymeth.2015.07.004

[26] Kurcinski, M. et al. CABS-dock web server for the flexible docking of peptides to proteins without prior knowledge of the binding site. *Nucleic Acids Res* **43**, W419–W424 (2015). 25943545 10.1093/nar/gkv456 PMC4489223

[27] Adasme, M. F. et al. PLIP 2021: expanding the scope of the protein–ligand interaction profiler to DNA and RNA. *Nucleic Acids Res* **49**, W530–W534 (2021). 33950214 10.1093/nar/gkab294 PMC8262720

[28] Park, H. et al. GV1001 modulates neuroinflammation and improves memory and behavior through the activation of gonadotropin-releasing hormone receptors in a triple transgenic Alzheimer’s disease mouse model. *Brain Behav Immun* **115**, 295–307 (2024). 37884161 10.1016/j.bbi.2023.10.021

[29] Liu, Y. et al. Disrupted blood–brain barrier in 5× FAD mouse model of Alzheimer’s disease can be mimicked and repaired in vitro with neural stem cell-derived exosomes. *Biochem Biophys Res Commun* **525**, 192–196 (2020). 10.1016/j.bbrc.2020.02.074 32081424

[30] Oblak, A. L. et al. Comprehensive evaluation of the 5XFAD mouse model for preclinical testing applications: a MODEL-AD study. *Front Aging Neurosci* **13**, 713726 (2021). 34366832 10.3389/fnagi.2021.713726 PMC8346252

[31] Zhong, L. et al. Soluble TREM2 ameliorates pathological phenotypes by modulating microglial functions in an Alzheimer’s disease model. *Nat Commun* **10**, 1365 (2019). 30911003 10.1038/s41467-019-09118-9 PMC6433910

[32] Zhang, Y. -w et al. APP processing in Alzheimer’s disease. *Mol Brain* **4**, 1–13 (2011). 21214928 10.1186/1756-6606-4-3 PMC3022812

[33] Vailati-Riboni, M. et al. Dietary fiber as a counterbalance to age-related microglial cell dysfunction. *Front Nutr* **9**, 835824 (2022). 35360677 10.3389/fnut.2022.835824 PMC8964049

[34] Slota, J. A. et al. Dysregulation of neuroprotective astrocytes, a spectrum of microglial activation states, and altered hippocampal neurogenesis are revealed by single-cell RNA sequencing in prion disease. *Acta Neuropathol Commun* **10**, 161 (2022). 36352465 10.1186/s40478-022-01450-4 PMC9647949

[35] Yin, Z. et al. Identification of a protective microglial state mediated by miR-155 and interferon-γ signaling in a mouse model of Alzheimer’s disease. *Nat Neurosci* **26**, 1196–1207 (2023). 37291336 10.1038/s41593-023-01355-y PMC10619638

[36] Yin, Z. et al. APOE4 impairs the microglial response in Alzheimer’s disease by inducing TGFbeta-mediated checkpoints. *Nat Immunol* **24**, 1839–1853 (2023). 37749326 10.1038/s41590-023-01627-6 PMC10863749

[37] Morisaki, Y. et al. LAG-3 expression in microglia regulated by IFN-gamma/STAT1 pathway and metalloproteases. *Front Cell Neurosci* **17**, 1308972 (2023). 38026700 10.3389/fncel.2023.1308972 PMC10663313

[38] Brandao, W. et al. Inhaled xenon modulates microglia and ameliorates disease in mouse models of amyloidosis and tauopathy. *Sci Transl Med* **17**, eadk3690 (2025). 39813317 10.1126/scitranslmed.adk3690 PMC13006031

[39] Fang, Y. et al. The adhesion and migration of microglia to β-amyloid (Aβ) is decreased with aging and inhibited by Nogo/NgR pathway. *J Neuroinflamm* **15**, 1–16 (2018). 10.1186/s12974-018-1250-1 PMC6054753 30029608

[40] Pan, R.-Y. et al. Sodium rutin ameliorates Alzheimer’s disease-like pathology by enhancing microglial amyloid-β clearance. *Sci Adv* **5**, eaau6328 (2019). 30820451 10.1126/sciadv.aau6328 PMC6393001

[41] Krasemann, S. et al. The TREM2–APOE pathway drives the transcriptional phenotype of dysfunctional microglia in neurodegenerative diseases. *Immunity* **47**, 566–581.e9 (2017). 28930663 10.1016/j.immuni.2017.08.008 PMC5719893

[42] Giannone, G. et al. Lamellipodial actin mechanically links myosin activity with adhesion-site formation. *Cell* **128**, 561–575 (2007). 17289574 10.1016/j.cell.2006.12.039 PMC5219974

[43] Ridley, A. Life at the leading edge. *Cell* **145**, 1012–1022 (2011). 21703446 10.1016/j.cell.2011.06.010

[44] Bolmont, T. et al. Dynamics of the microglial/amyloid interaction indicate a role in plaque maintenance. *J Neurosci* **28**, 4283–4292 (2008). 18417708 10.1523/JNEUROSCI.4814-07.2008 PMC3844768

[45] Bragina, M. E. et al. The SwissSimilarity 2021 web tool: novel chemical libraries and additional methods for an enhanced ligand-based virtual screening experience. *Int J Mol Sci* **23**, 811 (2022). 35054998 10.3390/ijms23020811 PMC8776004

[46] Nokkari, A. et al. Implication of the kallikrein-kinin system in neurological disorders: quest for potential biomarkers and mechanisms. *Prog Neurobiol* **165**, 26–50 (2018). 29355711 10.1016/j.pneurobio.2018.01.003 PMC6026079

[47] Marceau, F. et al. Bifunctional ligands of the bradykinin B2 and B1 receptors: an exercise in peptide hormone plasticity. *Peptides* **105**, 37–50 (2018). 29802875 10.1016/j.peptides.2018.05.007

[48] Arakawa, K. & Maruta, H. Ability of kallikrein to generate angiotensin II-like pressor substance and a proposed kinin–tensin enzyme system. *Nature* **288**, 705–706 (1980). 6905924 10.1038/288705a0

[49] Asraf, K. et al. Involvement of the bradykinin B1 receptor in microglial activation: in vitro and in vivo studies. *Front Endocrinol* **8**, 82 (2017). 10.3389/fendo.2017.00082 PMC5396024 28469598

[50] Asraf, K. et al. Differential effect of intranasally administrated kinin B1 and B2 receptor antagonists in Alzheimer’s disease mice. *Biol Chem* **397**, 345–351 (2016). 26556847 10.1515/hsz-2015-0219

[51] Fleisher-Berkovich, S. et al. Distinct modulation of microglial amyloid β phagocytosis and migration by neuropeptides. *J Neuroinflamm* **7**, 1–13 (2010). 10.1186/1742-2094-7-61 PMC2964654 20937084

[52] Ifuku, M. et al. Bradykinin-induced microglial migration mediated by B1-bradykinin receptors depends on Ca^2+^ influx via reverse-mode activity of the Na^+^/Ca^2+^ exchanger. *J Neurosci* **27**, 13065–13073 (2007). 18045900 10.1523/JNEUROSCI.3467-07.2007 PMC6673405

[53] Rex, D. et al. A modular map of bradykinin-mediated inflammatory signaling network. *J Cell Commun Signal* **16**, 301–310 (2022). 34714516 10.1007/s12079-021-00652-0 PMC8554507

[54] Senoo, H. et al. Hetero-oligomerization of Rho and Ras GTPases connects GPCR activation to mTORC2-AKT signaling. *Cell Rep* **33**, 108427 (2020). 10.1016/j.celrep.2020.108427 PMC7716479 33238110

[55] Srivastava, S. et al. Bradykinin regulates osteoblast differentiation by Akt/ERK/NFκB signaling axis. *J Cell Physiol* **229**, 2088–2105 (2014). 24825463 10.1002/jcp.24668

[56] Laplante, M. & Sabatini, D. M. mTOR signaling in growth control and disease. *Cell* **149**, 274–293 (2012). 22500797 10.1016/j.cell.2012.03.017 PMC3331679

[57] Wullschleger, S. et al. TOR signaling in growth and metabolism. *Cell* **124**, 471–484 (2006). 16469695 10.1016/j.cell.2006.01.016

[58] Saxton, R. A. & Sabatini, D. M. mTOR signaling in growth, metabolism, and disease. *Cell* **168**, 960–976 (2017). 28283069 10.1016/j.cell.2017.02.004 PMC5394987

[59] Chantaravisoot, N. et al. mTORC2 interactome and localization determine aggressiveness of high-grade glioma cells through association with gelsolin. *Sci Rep* **13**, 7037 (2023). 37120454 10.1038/s41598-023-33872-y PMC10148843

[60] Rispal, D. et al. Target of rapamycin complex 2 regulates actin polarization and endocytosis via multiple pathways. *J Biol Chem* **290**, 14963–14978 (2015). 25882841 10.1074/jbc.M114.627794 PMC4463442

[61] Johnson, J. L. et al. TORC2: a novel target for treating age-associated memory impairment. *Sci Rep* **5**, 1–9 (2015). 10.1038/srep15193 PMC4614817 26489398

[62] Weichhart, T. et al. Regulation of innate immune cell function by mTOR. *Nat Rev Immunol* **15**, 599–614 (2015). 26403194 10.1038/nri3901 PMC6095456

[63] Fleming, A. et al. Chemical modulators of autophagy as biological probes and potential therapeutics. *Nat Chem Biol* **7**, 9–17 (2011). 21164513 10.1038/nchembio.500

[64] Jacinto, E. et al. SIN1/MIP1 maintains rictor-mTOR complex integrity and regulates Akt phosphorylation and substrate specificity. *Cell* **127**, 125–137 (2006). 16962653 10.1016/j.cell.2006.08.033

[65] Kim, D.-H. et al. mTOR interacts with raptor to form a nutrient-sensitive complex that signals to the cell growth machinery. *Cell* **110**, 163–175 (2002). 12150925 10.1016/s0092-8674(02)00808-5

[66] Benmamar-Badel, A. et al. Protective microglial subset in development, aging, and disease: lessons from transcriptomic studies. *Front Immunol* **11**, 430 (2020). 32318054 10.3389/fimmu.2020.00430 PMC7147523

[67] Ennerfelt, H. et al. SYK coordinates neuroprotective microglial responses in neurodegenerative disease. *Cell* **185**, 4135–4152.e22 (2022). 36257314 10.1016/j.cell.2022.09.030 PMC9617784

[68] Alexander, G. C. et al. Evaluation of aducanumab for Alzheimer disease: scientific evidence and regulatory review involving efficacy, safety, and futility. *JAMA* **325**, 1717–1718 (2021). 33783469 10.1001/jama.2021.3854

[69] Van Dyck, C. H. et al. Lecanemab in early Alzheimer’s disease. *New Engl J Med* **388**, 9–21 (2023). 36449413 10.1056/NEJMoa2212948

[70] Sims, J. R. et al. Donanemab in early symptomatic Alzheimer disease: the TRAILBLAZER-ALZ 2 randomized clinical trial. *JAMA* **330**, 512–527 (2023). 37459141 10.1001/jama.2023.13239 PMC10352931

[71] Solopova, E. et al. Fatal iatrogenic cerebral β-amyloid-related arteritis in a woman treated with lecanemab for Alzheimer’s disease. *Nat Commun* **14**, 8220 (2023). 38086820 10.1038/s41467-023-43933-5 PMC10716177

[72] Reardon, S. Alzheimer’s drug donanemab: what promising trial means for treatments. *Nature* **617**, 232–233 (2023). 37142723 10.1038/d41586-023-01537-5

[73] Ji, B. et al. The dual role of kinin/kinin receptors system in Alzheimer’s disease. *Front Mol Neurosci* **12**, 234 (2019). 31632239 10.3389/fnmol.2019.00234 PMC6779775

[74] Prediger, R. et al. Genetic deletion or antagonism of kinin B1 and B2 receptors improves cognitive deficits in a mouse model of Alzheimer’s disease. *Neuroscience* **151**, 631–643 (2008). 18191900 10.1016/j.neuroscience.2007.11.009

[75] Lacoste, B. et al. Cognitive and cerebrovascular improvements following kinin B1 receptor blockade in Alzheimer’s disease mice. *J Neuroinflamm* **10**, 1–18 (2013). 10.1186/1742-2094-10-57 PMC3710240 23642031

[76] Noda, M. et al. Anti-inflammatory effects of kinins via microglia in the central nervous system. *Biol Chem* **387**, 167–171 (2006). 16497148 10.1515/BC.2006.022

[77] Noda, M. et al. Multifunctional effects of bradykinin on glial cells in relation to potential anti-inflammatory effects. *Neurochem Int* **51**, 185–191 (2007). 17669557 10.1016/j.neuint.2007.06.017

[78] Passos, G. F. et al. The bradykinin B1 receptor regulates Aβ deposition and neuroinflammation in Tg-SwDI mice. *Am J Pathol* **182**, 1740–1749 (2013). 23470163 10.1016/j.ajpath.2013.01.021 PMC3644719

[79] Singh, P. K. et al. Increased plasma bradykinin level is associated with cognitive impairment in Alzheimer’s patients. *Neurobiol Dis* **139**, 104833 (2020). 32173555 10.1016/j.nbd.2020.104833 PMC7175647

[80] Srinivasan, K. et al. Alzheimer’s patient microglia exhibit enhanced aging and unique transcriptional activation. *Cell Rep* **31**, 107843 (2020) 10.1016/j.celrep.2020.107843 PMC7422733 32610143

[81] Gougat, J. et al. SSR240612 [(2R)-2-[((3R)-3-(1,3-benzodioxol-5-yl)-3-[[(6-methoxy-2-naphthyl)sulfonyl]amino]propanoyl)amino]-3-(4-[[2R,6S)-2,6-dimethylpiperidinyl]methyl]phenyl)-*N*-isopropyl-*N*-methylpropanamide hydrochloride], a new nonpeptide antagonist of the bradykinin B1 receptor: biochemical and pharmacological characterization. *J Pharmacol Exp Ther* **309**, 661–669 (2004). 14747609 10.1124/jpet.103.059527

[82] Talbot, S. et al. Cellular localization of kinin B1 receptor in the spinal cord of streptozotocin-diabetic rats with a fluorescent [Nalpha-Bodipy]-des-Arg9-bradykinin. *J Neuroinflamm* **6**, 11 (2009). 10.1186/1742-2094-6-11 PMC2667487 19323833

[83] Campos, M. M. et al. Autoradiographic distribution and alterations of kinin B(2) receptors in the brain and spinal cord of streptozotocin-diabetic rats. *Synapse* **58**, 184–192 (2005). 16138314 10.1002/syn.20196

[84] Park, H.-H. et al. Neural stem cells injured by oxidative stress can be rejuvenated by GV1001, a novel peptide, through scavenging free radicals and enhancing survival signals. *Neurotoxicology* **55**, 131–141 (2016). 27265016 10.1016/j.neuro.2016.05.022

[85] Park, H.-H. et al. The novel vaccine peptide GV1001 effectively blocks β-amyloid toxicity by mimicking the extra-telomeric functions of human telomerase reverse transcriptase. *Neurobiol Aging* **35**, 1255–1274 (2014). 24439482 10.1016/j.neurobiolaging.2013.12.015

[86] Anderson, R. M. et al. Why do so many clinical trials of therapies for Alzheimer’s disease fail? *Lancet* **390**, 2327–2329 (2017). 29185425 10.1016/S0140-6736(17)32399-1

[87] Schott, J. M. et al. Unsuccessful trials of therapies for Alzheimer’s disease. *Lancet* **393**, 29 (2019). 30614456 10.1016/S0140-6736(18)31896-8

[88] Kumari, S. et al. Apoptosis in Alzheimer’s disease: insight into the signaling pathways and therapeutic avenues. *Apoptosis* **1**, 15 (2023). 10.1007/s10495-023-01848-y 37186274

[89] Motori, E. et al. Inflammation-induced alteration of astrocyte mitochondrial dynamics requires autophagy for mitochondrial network maintenance. *Cell Metab* **18**, 844–859 (2013). 24315370 10.1016/j.cmet.2013.11.005

[90] Yang, F. et al. Reactive astrocytes secrete the chaperone HSPB1 to mediate neuroprotection. *Sci Adv* **10**, eadk9884 (2024). 38507480 10.1126/sciadv.adk9884 PMC10954207

[91] Agnew-Svoboda, W. et al. A genetic tool for the longitudinal study of a subset of post-inflammatory reactive astrocytes. *Cell Rep Methods* **2**, 100276 (2022). 36046623 10.1016/j.crmeth.2022.100276 PMC9421582

[92] Sirko, S. et al. Injury-specific factors in the cerebrospinal fluid regulate astrocyte plasticity in the human brain. *Nat Med* **29**, 3149–3161 (2023). 38066208 10.1038/s41591-023-02644-6 PMC10719094

[93] Jumper, J. et al. Highly accurate protein structure prediction with AlphaFold. *Nature* **596**, 583–589 (2021). 34265844 10.1038/s41586-021-03819-2 PMC8371605

