# Supplementary material for: A human telomerase reverse transcriptase-derived peptide GV1001 rescues neurodegeneration in a mouse model of Alzheimer disease
Source: Exp Mol Med. 2026 Jun 3;58(6):1789–805. doi: 10.1038/s12276-026-01729-9 (PMC13323720; doi:10.1038/s12276-026-01729-9)
Supplement: Supplementary file 1 — Supplementary Information [file 12276_2026_1729_MOESM1_ESM.pdf]

Supplementary Information

Supplementary Materials and Methods

Antibodies and reagents

Commercially available antibodies and reagents used in this study were listed in Supplementary Table 2.

Behavior test

On day 1, mice were placed in a square-shaped arena (40 x 40 cm^2) for an open filed test (OFT) for 20 min to measure locomotion activity. On days 2, object recognition test (ORT) was performed in the same arena. In trial sessions, mice were allowed to explore identical objects for 10 min. After 1 h, one of the objects was replaced with a new object. The discrimination index was defined as $(T_n - T_f) / (T_n + T_f)$; where T_n = time spent in the vicinity of a new object, T_f = time spent in the vicinity of a familiar object. On day 3, mice were placed in a Y-shaped maze with three arms and allowed to explore for 6 min. Spontaneous alternation was defined as (number of three consecutive arm entries) / (total arm entry-2) × 100. All experimental groups were tested with their littermate WT control. After the behavior test, mice were anesthetized and brains were isolated and used for the experiments.

Immunohistochemistry

Mice were anesthetized with avertin and then perfused with PBS containing heparin (20 U/ml). Brains were post-fixed in 4% paraformaldehyde (PFA) for 48 h, and transferred to 30% sucrose until they sank to the bottom of the tube. Antigen retrieval was performed by heating the sections in 10 mM sodium citrate, 0.05% Tween 20 (pH 6.0) at 95°C for 10 min. The sections were then blocked with blocking buffer (5% BSA, 0.1% Triton X-100 in PBS) for 1 h. After washing with PBS, sections were labeled at 4°C overnight in antibody dilution buffer (2% BSA, 0.1% Triton X-100 in PBS) containing primary antibodies. Subsequently, sections were washed 3 times with PBST (0.05% Tween 20 in PBS), incubated with fluorescently conjugated secondary antibodies (diluted 1:500) at room temperature for 2 h and mounted. Images were acquired with an LSM700 confocal microscope (Carl Zeiss) and analyzed with ZEN software (Carl Zeiss).

Golgi-Cox staining

Mouse blood was removed through the femoral artery and the brain was isolated. Brains were washed in distilled water to remove remaining blood and put in impregnation solution from a Hito Golgi-Cox OptimStain kit (Hitobiotec Corp.) as in the manufacturer's instruction.

Immunocytochemistry

Primary cultured microglia were fixed with 4% PFA for 10 min and permeabilized with 0.1% Triton X-100 for 10 min. For F-actin staining with phalloidin, microglia were washed with pre-warmed PBS, fixed with 3.7% PFA for 15 min, and permeabilized with 0.1% Triton X-100 for 15 min. Cells were blocked with antibody diluent reagent

solution (Thermo Fisher Scientific) for 30 min and incubated with primary antibodies at room temperature for 2 h, rinsed 3 times with PBS containing 0.1% Tween-20 and incubated with appropriate fluorescent dye-conjugated secondary antibodies (1:500) at room temperature for 1 h. Nuclei were stained with Hoechst 33342 (Invitrogen). Images were obtained using an LSM700 confocal microscope.

Western blot analysis

Hippocampus and cortex were dissected, directly deep-frozen in liquid nitrogen and stored at -80°C until use. Each sample was homogenized in 300 μl radioimmunoprecipitation assay buffer (Sigma-Aldrich) with 1× protease and phosphatase inhibitor cocktails (Thermo Fisher Scientific) by using a glass homogenizer (Chamlide CMB). The homogenates were centrifuged and each supernatant was collected. Samples were separated by SDS-gel electrophoresis and electro-transferred membranes were then incubated with primary antibodies overnight at 4°C, washed and incubated with peroxidase-conjugated secondary antibodies at room temperature for 2 h. Horseradish peroxidase–conjugated ACTB/β-actin (Santa Cruz Biotechnology) as a housekeeping protein was used for normalization. Chemiluminescence detection was performed to analyze the protein bands of interest. The blots were quantified using ImageJ (National Institutes of Health).

Prussian blue staining

Iron (Fe)-labeled GV1001 was generated by PEPTRon (Korea). Mice were subcutaneously injected with Fe-GV1001 (4 mg/kg) and sacrificed 2 h after injection. Fe- GV1001 was

detected with a Prussian blue staining kit (Abcam) according to the manufacturer's instructions.

TUNEL assay

Frozen sections were incubated with digestion buffer (0.2% Triton X-100, 0.1% Tween-20 in PBS) for 30 min, washed 3 times with PBST and incubated in 3% H_2O_2 for 10 min to block endogenous peroxidase activity. After washing with PBST 3 times, TUNEL assay was performed with a DeadEnd TUNEL assay kit (Promega) as in the manufacturer's instructions. Sections were further incubated with IBA1 and 6E10 antibodies to detect $TUNEL^+$ microglia near plaque.

Proximity ligation assay

FITC-fAβ_{1-42} (0.3 μM) was incubated with primary microglia for 2 h. After fixation with 4% PFA and permeabilization with 0.1% Triton X-100, cells were incubated with mouse anti-Aβ (Cell Signaling Technology) and rabbit anti-LAMP2 antibodies (Sigma-Aldrich) and then with a pair of PLA probes (Sigma-Aldrich); probe ligation, signal amplification (Sigma-Aldrich), and mounting (Sigma-Aldrich) were performed according to the manufacturer's instructions. PLA signals were obtained using an LSM700 confocal microscope.

Quantitative RT-PCR

With RNA obtained from LCM samples, cDNA was synthesized using SuperScriptTMIV First-Strand Synthesis System (Invitrogen). RNA was purified from primary microglia using the QIAzol Lysis Reagent (Qiagen), and cDNA was synthesized using the ImProm-II Reverse Transcriptase kit (Promega) and oligo-dT primers (Promega). TOPreal qPCR 2× PreMIX (SYBR Green with low ROX; Enzynomics) containing nTaq-HOT DNA polymerase was used for qRT-PCR, which was performed in a CFX96 Real-Time System (Bio-Rad). The following primers were used as listed in Supplementary Table 3.

Preprocessing and Quality Control

Raw sequencing data were processed using the CellRanger pipeline (v8.0.0) with the mm10 reference genome (refdata-gex-mm10-2020-A). Cells with fewer than 200 total unique molecular identifiers (UMIs) or more than 20% mitochondrial gene content were excluded. Given the transcriptomic heterogeneity and cellular stress associated with aged 5xFAD brain tissue, relatively permissive thresholds were applied to retain microglial populations with low RNA content. Empty droplets were identified and removed using the DropletUtils package (false discovery rate (FDR) ≤ 0.05), and per-cell QC metrics were calculated using the scuttle package.

Normalization and Clustering

The filtered expression matrix was processed using the Seurat package (v4.3.0) in R (v4.4.3). Data were normalized using the LogNormalize method and subsequently scaled. The top 1,000 highly variable genes were selected for principal component analysis (PCA), and the first 17 principal components were used for graph-based clustering and dimensionality reduction.

Uniform Manifold Approximation and Projection (UMAP) was applied for two-dimensional visualization of cell states. Cell types and microglial subtypes were manually annotated using canonical marker genes, including *Apoe* and *Axl* to distinguish DAM1 and DAM2.

Differential Expression Analysis

Cluster-specific marker genes were identified using the Wilcoxon rank-sum test, as implemented in Seurat's FindMarkers function, and results were adjusted for multiple testing using the Benjamini–Hochberg method (FDR < 0.05). Volcano plots were generated to visualize differentially expressed genes (DEGs) between GV1001 and saline groups, as well as within microglial subpopulations (homeostatic, DAM1, DAM2).

Pseudotime Analysis

Pseudotime trajectory analysis was conducted using the Monocle3 package (v1.2.9). Seurat objects containing homeostatic microglia, DAM1, and DAM2 subtypes were converted to CellDataSet objects using the as.cell_data_set function. Dimensionality reduction was performed using UMAP, followed by trajectory graph construction with learn_graphand pseudotime ordering using order_cells.

Gene Ontology Analysis

Gene ontology (GO) enrichment analysis was performed using the ClusterProfiler package (v4.6.0). DEGs from DAM1 and DAM2 microglia were analyzed for overrepresented biological processes, molecular functions, and cellular components. Among the GO categories,

cellular component (CC) terms were specifically selected and visualized to highlight compartmentalized changes associated with GV1001 treatment.

Gene Module Score Analysis

Module scores for migration and phagocytosis gene sets were calculated using the AddModuleScore function in Seurat (v4.3.0), based on a combined list of genes involved in microglial migration and phagocytosis. Scores were assessed across all microglia and within each subtype (homeostatic, DAM1, DAM2). Statistical significance between GV1001- and Saline groups was evaluated using unpaired two-sided t-tests.

Supplementary Figures

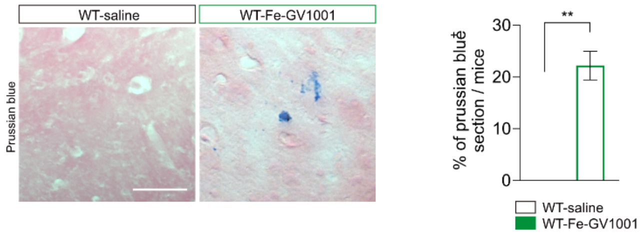

Supplementary Fig. 1. GV1001 crosses the blood–brain barrier. Mouse hippocampus was stained with Prussian blue 2 h after s.c. injection of iron-conjugated GV1001 (Fe-GV1001, 4 mg/kg). (*n* = 6-8 sections per mouse from 3 mice per group). Scale bar, 50 μm. Statistical comparison was conducted with unpaired *t*-test, $^{**}p<0.01$. Data are means ± SEM.

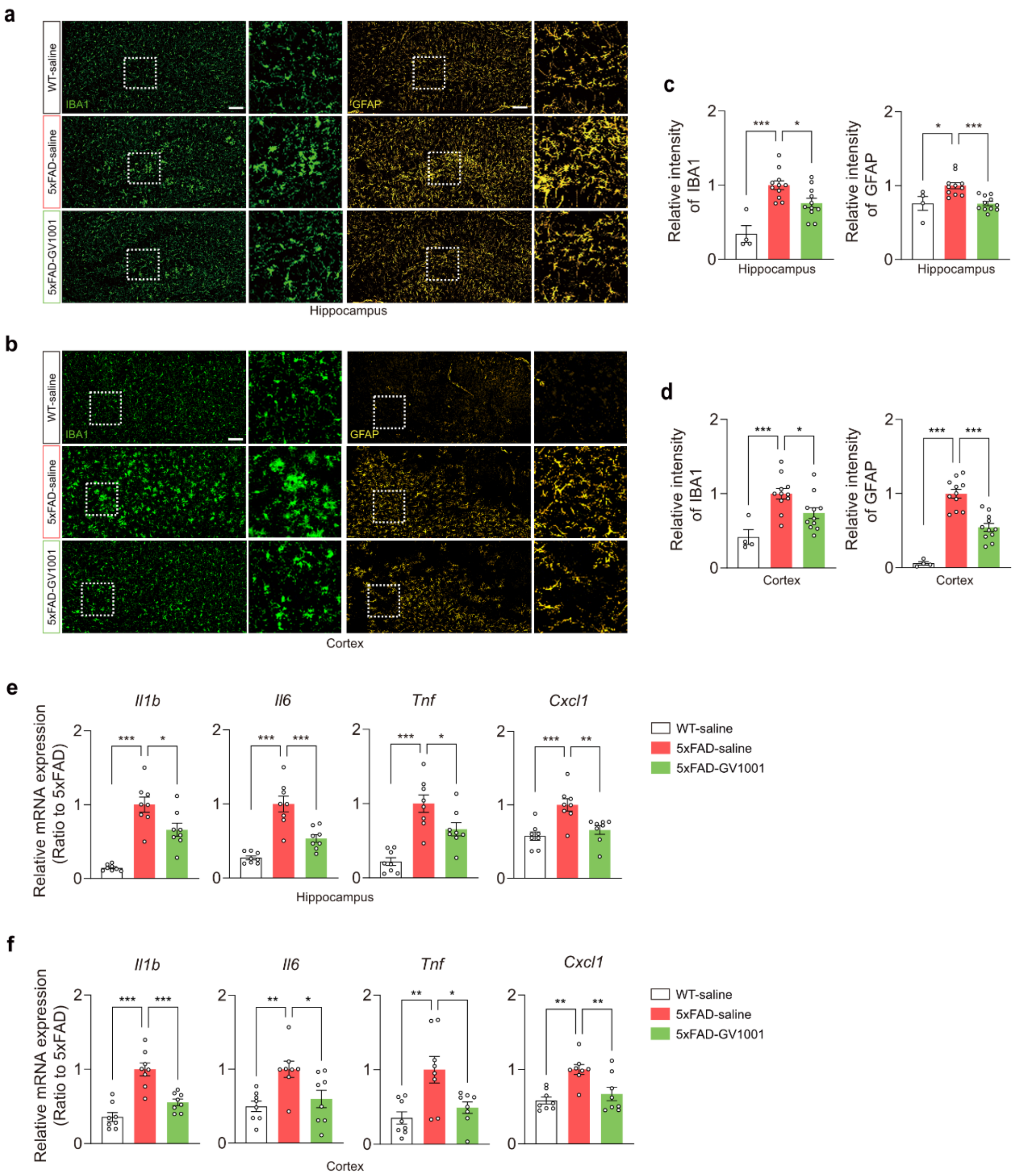

Supplementary Fig. 2. GV1001 reduces Aβ plaque burden and neuroinflammation in 5xFAD mice. a, b Representative immunofluorescence images obtained by staining with IBA1 and GFAP antibodies of the hippocampus (**a**) and cortex (**b**) of 8-9-month-old WT and 5xFAD mice. Scale bar, 100 μm. **c, d** Quantification of IBA1 (**c**) and GFAP intensities (**d**) of hippocampus and cortex (*n* = 4 mice for WT, *n* = 11 mice for 5xFAD-saline and 5xFAD-GV1001 groups). **e, f** Relative mRNA levels of cytokines in the hippocampus (**e**) and cortex (**f**)

were measured by qRT-PCR (*n* = 8 mice per group) and normalized to *Actb*. All statistical comparisons were conducted with one-way ANOVA followed by Tukey's multiple comparisons test, *p<0.05, **p<0.01, and ***p<0.001; ns, not significant. Data are means ± SEM.

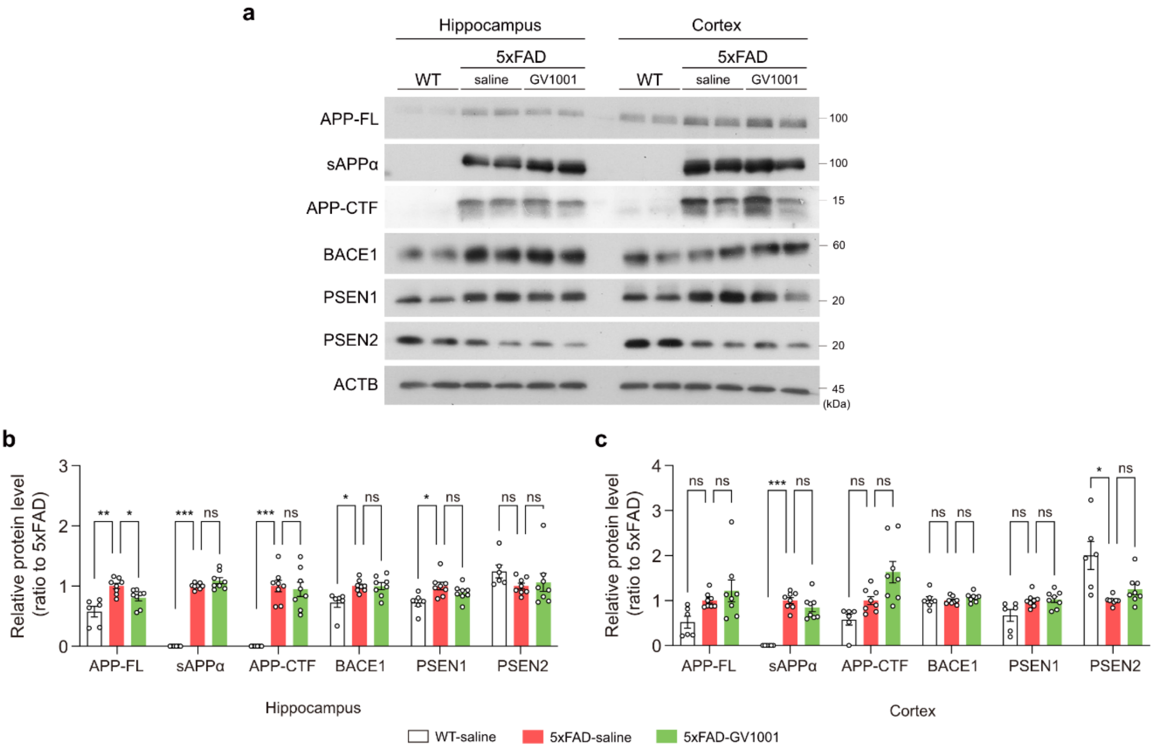

Supplementary Fig. 3. GV1001 does not alter APP processing. a Representative western blots of full-length APP (APP-FL), soluble APP alpha (sAPPα), APP carboxy-terminal fragment (APP-CTF), BACE1, PSEN1, and PSEN2 in the hippocampus and cortex of 8-9-month-old mice. **b, c** Mean protein levels in the hippocampus (**b**) and cortex (**c**) after normalization to ACTB. (n = 6 mice for WT, n = 8 mice for 5xFAD-saline and 5xFAD-GV1001 groups). All statistical comparisons were conducted with one-way ANOVA followed by Tukey's multiple comparisons test, *$p<0.05$, **$p<0.01$, and ***$p<0.001$; ns, not significant. Data are means ± SEM.

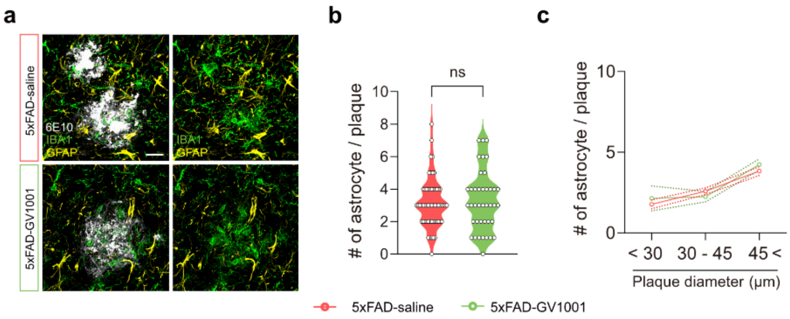

Supplementary Fig. 4. GV1001 does not promote astrocyte recruitment. a Representative immunofluorescence images obtained by staining with 6E10 and GFAP antibodies of the hippocampus of 8-9-month-old 5xFAD mice. Scale bar, 20 µm. **b** Number of astrocytes near Aβ plaques (n = 8 mice per group). **c** Analysis of the number of astrocytes per plaque depending on the size of Aβ plaques. All statistical comparisons were conducted with unpaired t-test, ns, not significant. Data are means ± SEM.

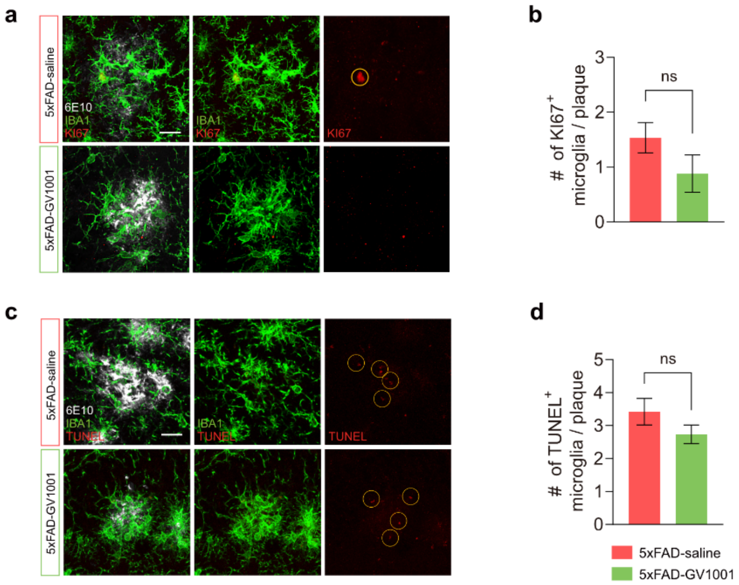

Supplementary Fig. 5. GV1001 does not alter proliferation or survival of microglia. a Representative immunofluorescence images obtained by staining with IBA1 and KI67 antibodies of the hippocampus of 8-9-month-old 5xFAD mice. Scale bar, 20 μm. **b** Quantification of $KI67^+$ microglia near large plaques in the hippocampus ($n = 5$ mice per group). **c** Representative immunofluorescence images obtained by staining with IBA1 antibody and TUNEL assay in the hippocampus of 8-9-month-old 5xFAD mice. Scale bar, 20 μm. **d** Quantification of $TUNEL^+$ microglia near large plaques in the hippocampus ($n = 5$ mice per group). Yellow circles indicate $KI67^+$ or $TUNEL^+$ signals co-localized with microglia near large plaques. All statistical comparisons were conducted with unpaired *t*-test. ns, not significant. Data are means ± SEM.

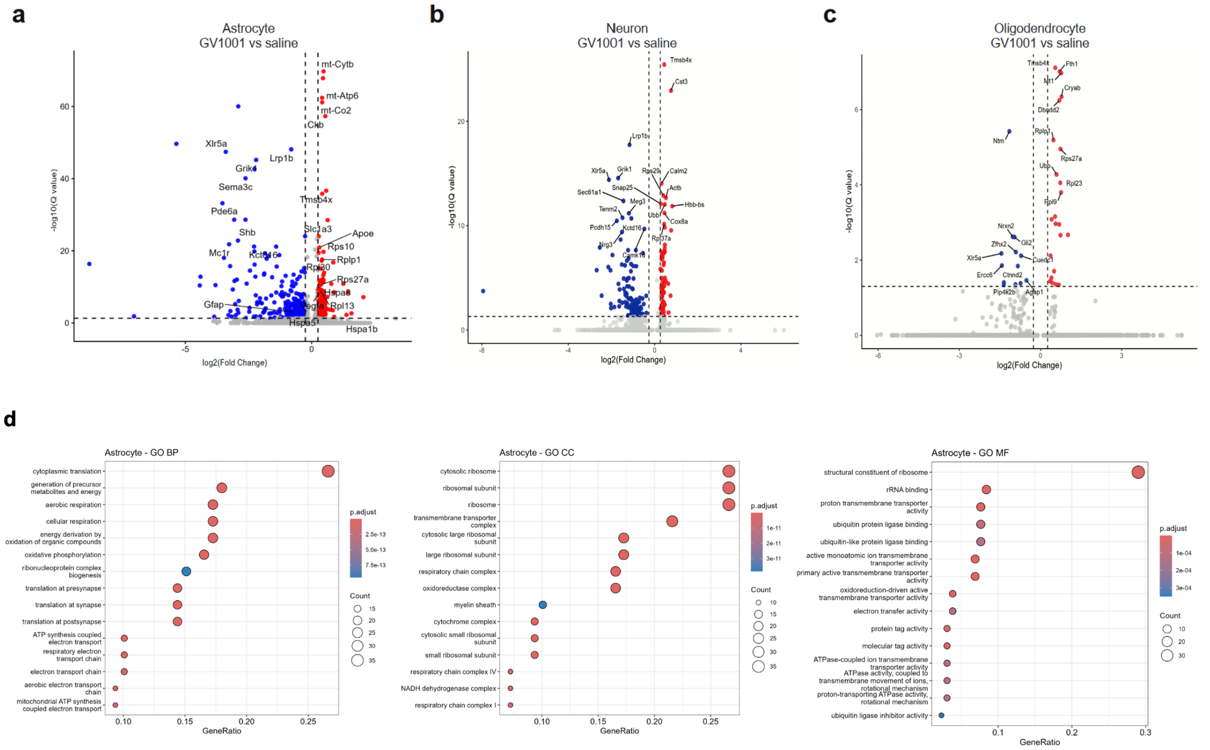

Supplementary Fig. 6. Volcano plots of astrocytes, neurons, and oligodendrocytes. a-c Volcano plots showing DEGs in astrocytes, neurons, and oligodendrocytes between GV1001-injected and saline-injected 5xFAD mice (*q-value* < 0.05). **d** GO analysis in astrocytes (p < 0.05, FDR corrected using Benjamini-Hochberg method).

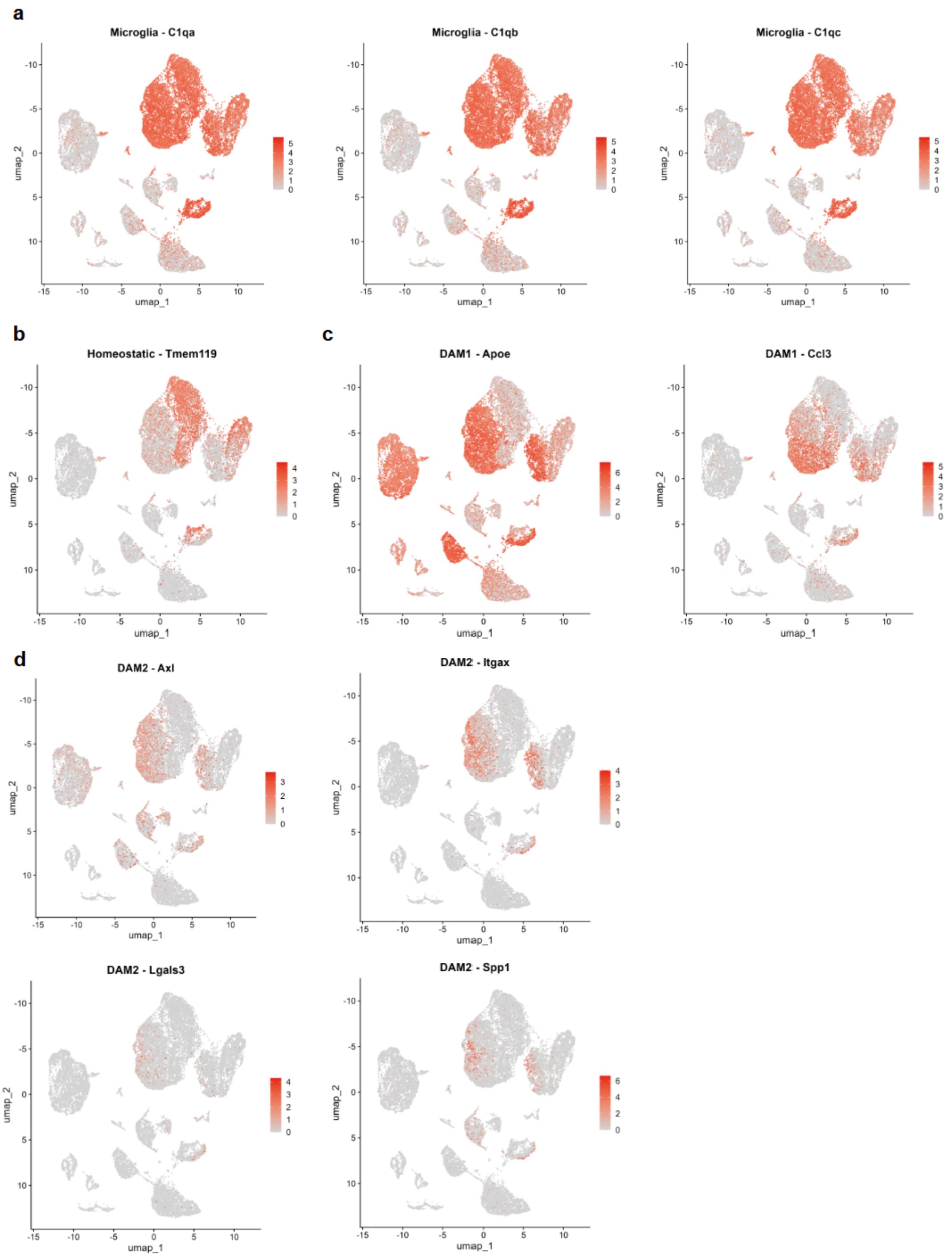

Supplementary Fig. 7. Expression of markers for homeostatic, DAM1, and DAM2 microglia. a Representative markers for microglia. **b-d** Representative markers for homeostatic, DAM1, and DAM2 microglia populations.

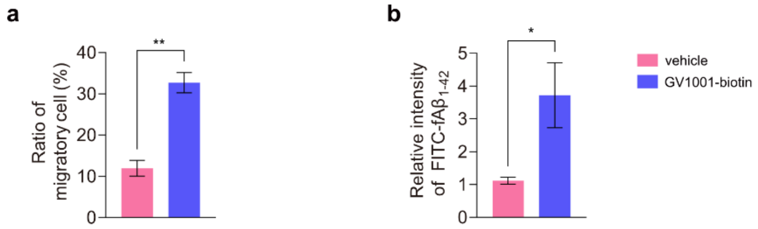

Supplementary Fig. 8. Biotinylation of GV1001 does not interfere with its efficacy. a Quantification of F-actin accumulation in lamellipodium in primary cultured microglia treated with biotin-GV1001 for 6 h (*n* = 62-73 cells from 4 experiments). **b** Quantification of FITC-fA$\beta_{1\text{-}42}$ intensity in primary cultured microglia treated with biotin-GV1001 for 6 h (*n* = 43-46 cells from 3 experiments). All statistical comparisons were conducted with unpaired *t*-test, *$p<0.05$, and **$p<0.01$; ns, not significant. Data are means ± SEM.

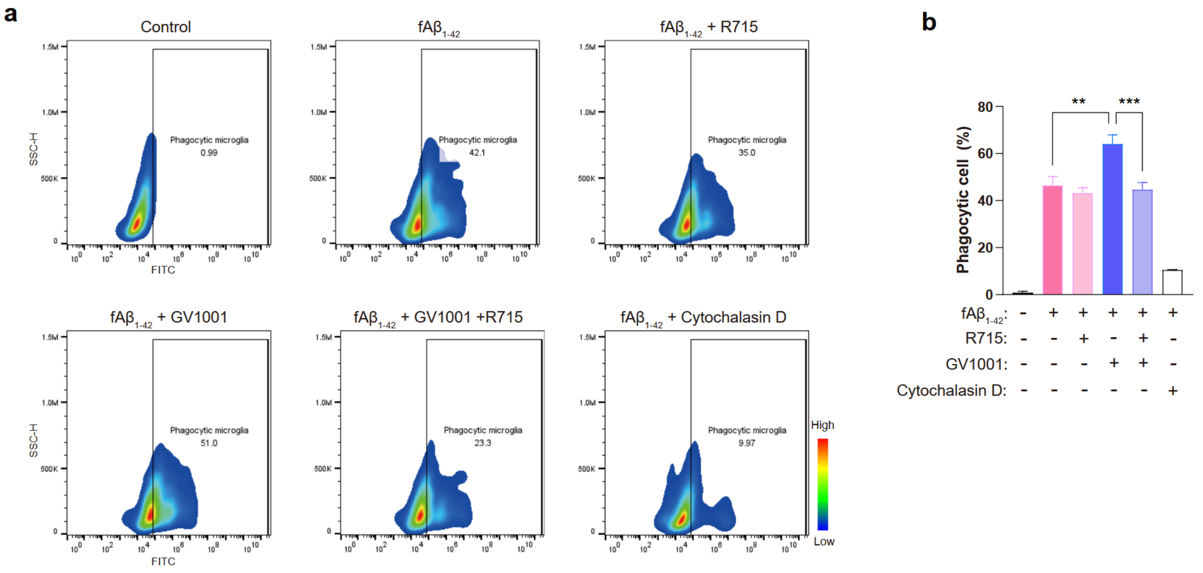

Supplementary Fig. 9. Flow cytometry-based phagocytosis assay in primary cultured microglia. a Representative flow cytometry plots of microglia for phagocytosis assay using FITC-fA$\beta_{1\text{-}42}$. **b** Quantification of phagocytosis of microglia after treatment with R715, GV1001, or cytochalasinD (n = 6). All statical comparisons were conducted with one-way ANOVA followed by Tukey's multiple comparisons test, **p<0.01, and ***p<0.001; ns, not significant. Data are means ± SEM.

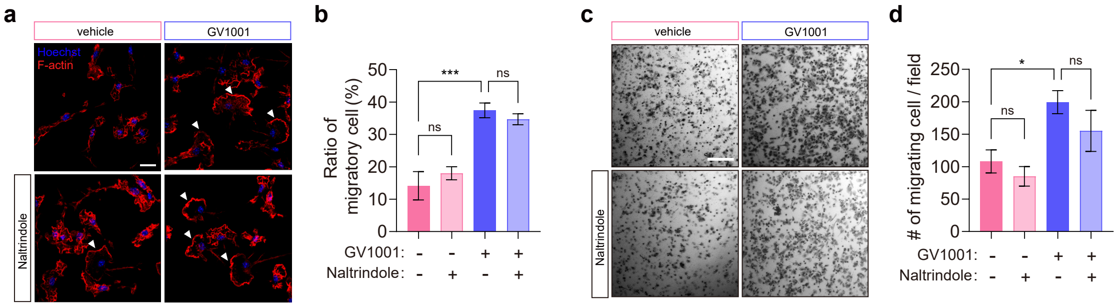

Supplementary Fig. 10. Naltrindole does not prevent GV1001-induced lamellipodium formation and migration of microglia. a Representative immunocytochemistry images of F-actin staining in primary cultured microglia treated with GV1001 and naltrindole (1 μM) for 6 h. Scale bar, 20 μm. **b** Quantification of the migratory microglia with F-actin accumulation in lamellipodium (n = 86-111 cells from 4 experiments). **c** Trans-well assay using primary cultured microglia treated with GV1001 and naltrindole. Scale bar, 20 μm. **d** Quantification of microglia migration ($n = 4$). All statistical comparisons were conducted with one-way ANOVA followed by Tukey's multiple comparisons test, *p<0.05, and ***p<0.001; ns, not significant. Data are means ± SEM.

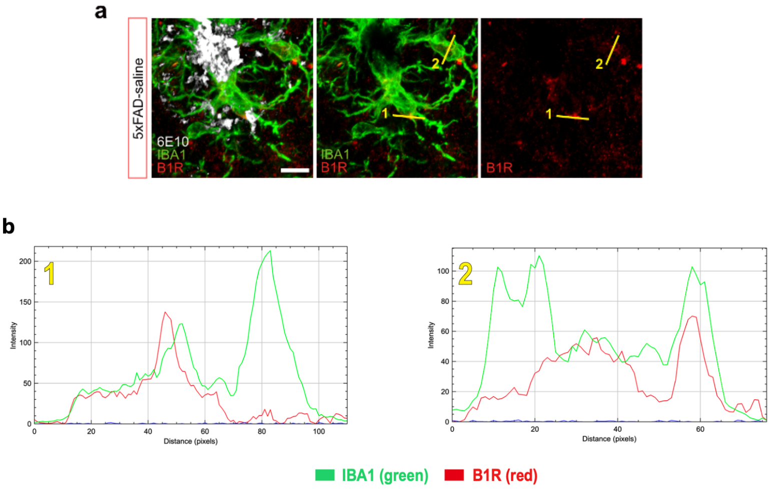

Supplementary Fig. 11. B1R is expressed in plaque-associated microglia in 5xFAD mice. **a** Representative immunofluorescence images obtained by co-staining with 6E10 (Aβ plaques), IBA1 (microglia), and B1R antibodies of the hippocampus of 8-9-month-old 5xFAD mice. Scale bar, 10 µm. **b** Colocalization analysis between IBA1 and B1R in microglia.

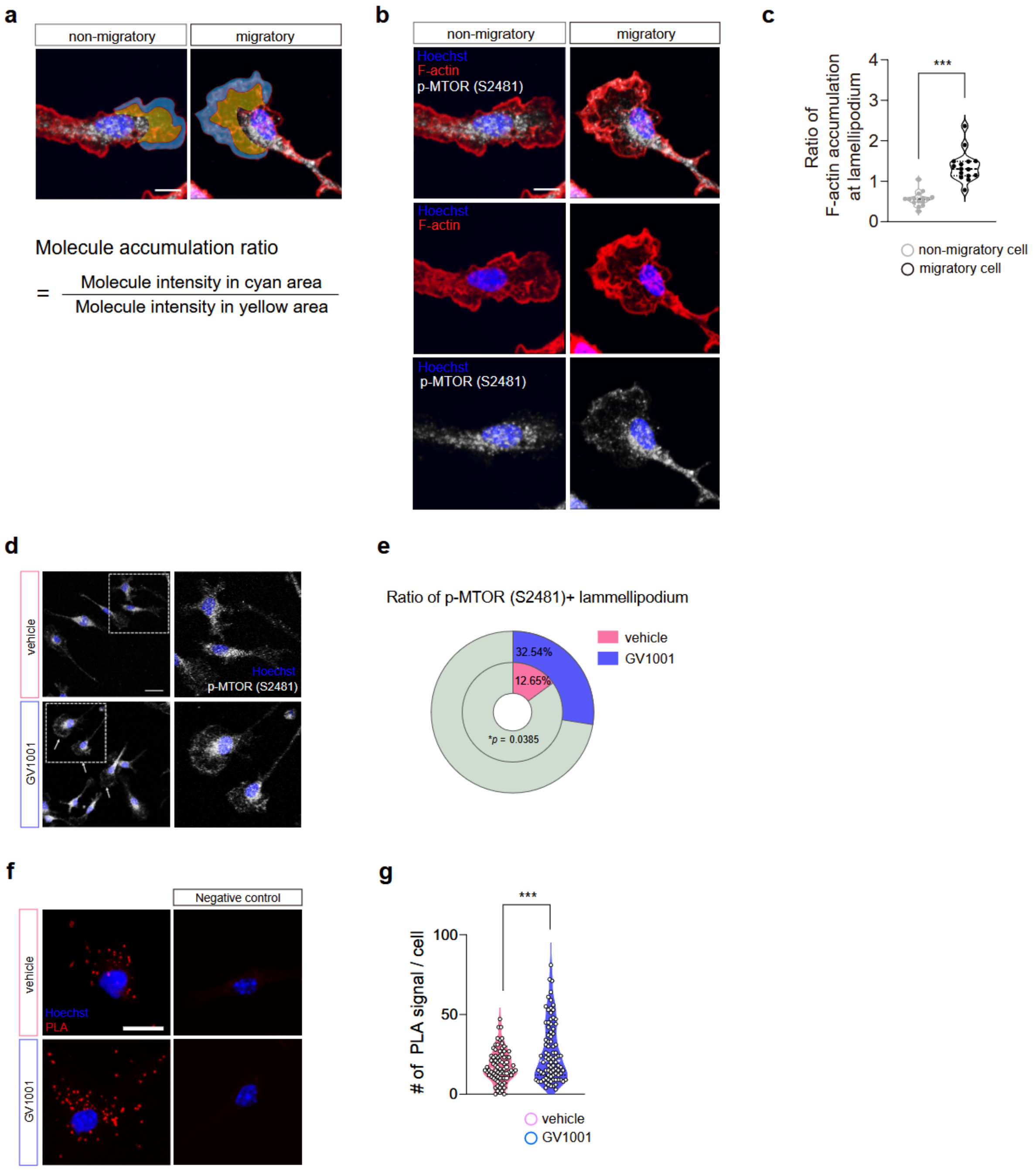

Supplementary Fig. 12. GV1001 promotes MTROC2 accumulation in lamellipodium and fAβ_{1-42} degradation. a Illustration for the analysis of molecule accumulation ratio in the lamellipodium. Signal intensities of the molecules of interest were measured and accumulation ratios were calculated following the formula. **b** Representative immunocytochemical images of staining for F-actin (phalloidin) and p-MTOR (S2481) in non-migratory and migratory

microglia (n = 15 cells from 3 experiments). Scale bar, 10 μm. **c** Accumulation of p-MTOR (S2481) in lamellipodium. **d** Representative immunocytochemical images of staining for p-MTOR (S2481) in microglia treated with GV1001 or saline for 6 h (vehicle) (n = 40-50 cells from 3 experiments). Scale bar, 20 μm. **e** Percentage of p-MTOR (S2481)$^+$ migratory microglia (n = 40-50 cells from 3 experiments). **f** Representative fluorescence images of proximity ligation assay (PLA) after treatment with GV1001 (1 μM) or saline (vehicle) for 24 h. Scale bar, 10 μm. **g** Number of PLA signals per cell (n = 65-71 cells from 3 experiments). All statistical comparisons were conducted with unpaired t-test, *p<0.05, and ***p<0.001; ns, not significant. Data are means ± SEM.

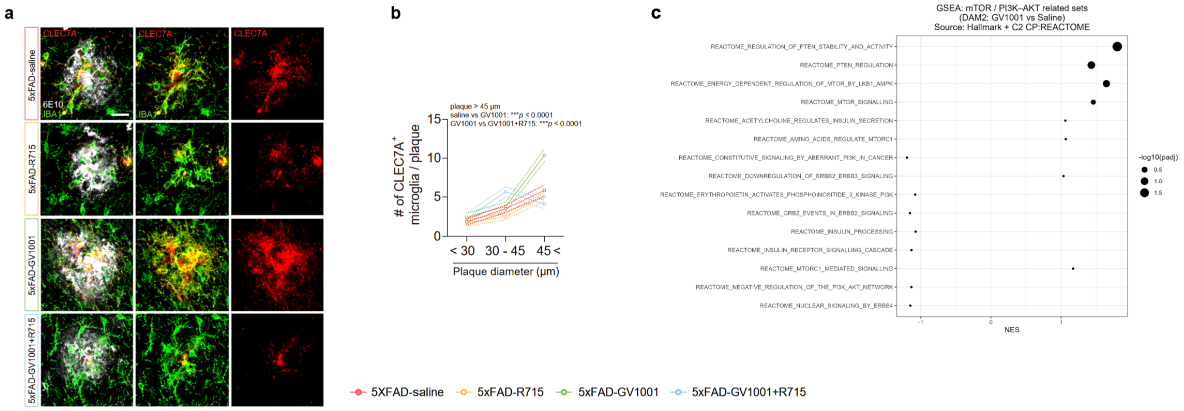

Supplementary Fig. 13. GV1001 increases CLEC7A$^+$ DAM2 in a B1R-dependent manner. **a** Representative immunofluorescence images obtained by co-staining with 6E10 (Aβ plaques), IBA1 (microglia), and CLEC7A antibodies of the hippocampus of 8-9-month-old 5xFAD mice. Scale bar, 10 μm. **b** Analysis of the number of CLEC7A+ microglia per plaque depending on the size of Aβ plaques (n = 5 mice per group). **c** Gene set enrichment analysis of DAM2 microglia comparing 5xFAD-GV1001 versus 5xFAD-saline, focusing on mTOR signaling pathway. All statistical comparisons were conducted with one-way ANOVA followed by Tukey's multiple comparisons test, ***p<0.001; ns, not significant. Data are means ± SEM.

Supplementary Table 1. Interaction residues between B1R and GV1001 (hydrogen bonds, hydrophobic interactions, and ionic bonds).

Hydrogen bonds

Index	Residue	AA	Distance H-A	Distance D-A	Donor angle	Protein donor?	Side chain	Donor atom	Acceptor atom	Ligand residue
1	100A	GLN	2.21	2.74	111.96	O	O	796 [Nam]	2878 [O.co2]	1GLU
2	114A	ASN	3.45	3.92	111.97	O	X	907 [Nam]	3001 [N3]	16LYS
3	114A	ASN	2.1	2.7	115.69	X	O	3001 [N3]	911 [O2]	12ARG
4	118A	LYS	2.86	3.32	108.02	O	O	940 [N3]	2965 [Ng^+]	12ARG
5	171A	PRO	3.3	3.75	109.93	X	X	2965 [Ng^+]	1371 [O2]	12ARG
6	176A	ARG	2.8	3.68	149.69	O	O	1413 [Ng^+]	2969 [Nam]	13PHE
7	176A	ARG	3.28	4.06	137.1	O	O	1414 [Ng^+]	2969 [Nam]	13PHE
8	202A	ARG	3.02	3.9	148.82	X	O	2966 [Ng^+]	1617 [Ng^+]	12ARG
9	202A	ARG	3	3.84	143.56	O	O	1619 [Ng^+]	2957 [O2]	11LEU
10	273A	GLU	2.52	2.91	105.21	O	O	2191 [O3]	2936 [O3]	9SER
11	273A	GLU	2.42	2.91	110.83	X	O	2936 [O3]	2191 [O3]	9SER
12	277A	GLN	2.17	3.08	153.72	O	O	2231 [Nam]	2932 [O2]	8THR
13	291A	ASP	2.61	3	103.61	X	X	2946 [Ng^+]	2351 [O2]	10ARG
14	298A	ASN	2.51	3.14	121.78	O	O	2399 [Nam]	2994 [O2]	15PRO

Hydrophobic interactions

Index	Residue	AA	Distance	Ligand atom	Protein atom	Ligand residue
1	33A	TRP	3.71	2875	249	1GLU
2	93A	TRP	3.54	2946	734	13PHE
3	93A	TRP	2.94	2999	732	16LYS
4	101A	PHE	3.82	2884	801	2ALA
5	117A	ILE	3.02	2991	930	15PRO
6	183A	ASP	3.78	2902	1461	4PRO
7	188A	ALA	3.86	2907	1500	5ALA
8	190A	ILE	3.71	2920	1512	7LEU
9	191A	LEU	2.95	2920	1522	7LEU
10	191A	LEU	3.89	2960	1520	12ARG
11	191A	LEU	3.67	2971	1521	13PHE
12	193A	LEU	3.07	2923	1537	7LEU
13	206A	LEU	2.96	2984	1651	14ILE
14	266A	TYR	2.94	2983	2125	14ILE
15	273A	GLU	3.13	2954	2189	11LEU
16	273A	GLU	3.62	2955	2188	11LEU
17	277A	GLN	3.74	2930	2228	8THR
18	294A	LEU	2.95	2985	2368	14ILE

Salt Bridges

Index	Residue	AA	Distance	Protein positive?	Ligand group	Ligand atoms	Ligand residue
1	37A	HIS	5.22	N/A	Carboxylate	2878, 2879	1GLU
2	291A	ASP	5.37	N/A	Guanidine	2944, 2946, 2947	10ARG

Supplementary Table 2. List of antibodies and reagents.

REAGENT or RESOURCE	SOURCE	IDENTIFIER
Antibodies		
Rabbit polyclonal anti-B1 Bradykinin Receptor (BDKRB1)	Alomone	Cat# ABR-011
Rabbit polyclonal anti-B2 Bradykinin Receptor (BDKRB1)	Alomone	Cat# ABR-012
Rat monoclonal anti-CD68	Abcam	Cat# ab53444
Rabbit polyclonal anti-KI67	Abcam	Cat# ab15580
Mouse monoclonal anti-β-Amyloid, 1-16 [6E10]	BioLegend	Cat# 803001
Mouse monoclonal anti- β-Amyloid, 1-16-HRP	BioLegend	Cat# 803012
Mouse monoclonal anti-PSD95	BD Biosciences	Cat# 610495
Rat monoclonal anti-CD16/CD32	BD Biosciences	Cat# 553142
Rabbit polyclonal anti-AKT1	Cell Signaling Technology	Cat# 9272
Rabbit polyclonal anti-APP	Cell Signaling Technology	Cat# 2452
Rabbit monoclonal anti-BACE1	Cell Signaling Technology	Cat# 5606
Mouse monoclonal anti-human β-Amyloid (D3D2N)	Cell Signaling Technology	Cat# 15126
Rabbit polyclonal anti-phospho-AKT1 (Ser473)	Cell Signaling Technology	Cat# 9271
Rabbit polyclonal anti-phospho-MTOR (Ser2481)	Cell Signaling Technology	Cat# 2974
Mouse monoclonal anti-phospho-p70S6 Kinase (Thr389)	Cell Signaling Technology	Cat# 9206
Rabbit monoclonal anti-Presenilin 1 (D39D1)	Cell Signaling Technology	Cat# 5643
Rabbit monoclonal anti-Presenilin 2 (D30G3)	Cell Signaling Technology	Cat# 9979
Rabbit monoclonal anti-p70S6 Kinase	Cell Signaling Technology	Cat# 2708
Chicken polyclonal anti-GFAP	Novus	Cat# NBP1-05198
Mouse monoclonal anti-β-actin-HRP (C4)	Santa Cruz	Cat# sc-47778 HRP
Rabbit polyclonal anti-LAMP2	Sigma-Aldrich	Cat# L0668
Goat polyclonal anti-IBA1	Wako Chemicals	Cat# 011-27991
Rabbit polyclonal anti-IBA1	Wako Chemicals	Cat# 019-19741
Rat monoclonal anti-mDectin-1-IgG	InvivoGen	Cat# mabg-mdect
Chemicals, Peptides, and Recombinant Proteins		
GV1001	GemVax&KAEL	N/A
Fe-GV1001	PEPTRon	N/A
GV1001-biotin	PEPTRon	N/A
Beta-Amyloid (1-42) 5-TAMRA-labeled Human	Anaspec	Cat# AS-60476
FITC-β-Ala-Amyloid β-Protein (1-42)	Bachem	Cat# 4033502
Alexa Fluor™ 555 Phalloidin	Invitrogen	Cat# A34055
Rapamycin	Enzo Life Sciences	Cat# BML-A275-0005
Cytochalasin D	Sigma	Cat# C2618
Naltrindole hydrochloride	Sigma	Cat# N115
Torin1	Tocris Bioscience	Cat# 4247
R715	Tocris Bioscience	Cat# 3407

HOE140	Tocris Bioscience	Cat# 3014
2x Laemmli Sample Buffer	Bio-Rad	Cat# 1610737
DMEM/High Glucose	Corning	Cat# 10-013-CV
TOPreal qPCR 2xPreMIX	Enzynomics	Cat# RT500M
RBC Lysis Buffer	eBioscience	Cat# 00-4333-57
RPMI1640	Gibco	Cat# 11875-093
HBSS, calcium, magnesium, no phenol red	Gibco	Cat# 14025092
DMEM/F12 (1:1)	Gibco	Cat# 11330-032
Trypsin-EDTA	Hyclone	Cat# SH30236.01
Fetal Bovine Serum	Hyclone	Cat# SH30919.03
Penicillin-Streptomycin 100X solution	Hyclone	Cat# SV30010
Hoechst 33342, Trihydrochloride, Trihydrate	Invitrogen	Cat# H3570
MACS LS Column	Miltenyibiotec	Cat# 130-042-401
MACS CD11b Microbead	Miltenyibiotec	Cat# 130-093-636
Oligo(dT)15 Primer	Promega	Cat# C1101
PEI MAX	Polysciences	Cat# 24765
Percoll	Sigma	Cat# P4937
Proteinase K	Sigma	Cat# P6556
Radioimmunoprecipitation assay (RIPA) buffer	Sigma	Cat# 89901
Diethyl pyrocarbonate (DEPC)	Sigma	Cat# D5758
Heparin sodium salt from porcine intestinal mucosa	Sigma	Cat# H3393
Hexadimethrine bromide	Sigma	Cat# H9268
Crystal Violet	SERVA	Cat# 27335.01
DNaseI	STEMCELL	Cat# 07900
Antibody diluent reagent solution	Thermo Fisher Scientific	Cat# 003218
Protease and phosphatase inhibitor cocktails	Thermo Fisher Scientific	Cat# 78440
BCA protein assay reagents	Thermo Fisher Scientific	Cat# 23225
RNase-Zap	Thermo Fisher Scientific	Cat# AM9782
SuperScript™ IV Reverse Transcriptase	Thermo Fisher Scientific	Cat# 18091050
DirectPCR Lysis Reagent (Tail)	Viagen Biotech	Cat# T101-T
Critical Commercial Assays		
PicoPure™ RNA Isolation Kit	Applied Biosystems	Cat# KIT0204
Hito Golgi-Cox OptimStain™ Kit	Hitobiotec	Cat# HTKNS1125
DeadEnd TUNEL assay Kit	Promega	Cat# G3250
ImProm-II Reverse Transcriptase Kit	Promega	Cat# A3803
Duolink® In Situ Detection Reagents Orange	Sigma	Cat# DUO92007
ImmPACT® DAB Substrate Kit, Peroxidase (HRP)	Vector Laboratories	Cat# SK-4105
Iron Stain Kit (Prussian Blue Stain)	Abcam	Cat# ab150674
Experimental Models: Cell Lines		
Lenti-X 293T Cell Line	Clonetech	Cat# 632180

Primary microglia from C57BL6	This paper	N/A
Oligonucleotides		
Primers for genotyping, see Supplementary Table2	This paper	N/A
Primers for qRT-PCR, see Supplementary Table2	This paper	N/A
Recombinant DNA		
Plasmid: PLKO.1-EGFP	This paper	N/A
shRNA: Target for *Bdkrb1*	Sigma	Cat# TRCN0000028130
shRNA: Target for *Bdkrb1*	Sigma	Cat# TRCN0000028128

Supplementary Table 3. Sequences of primers for genotyping and qRT-PCR.

Genotyping for 5xFAD	
Type	primer
Mutant Reverse	CGG GCC TCT TCG CTA TTA C
Common	ACC CCC ATG TCA GAG TTC CT
Wild type Reverse	TAT ACA ACC TTG GGG GAT GG

qRT-PCR		
Gene	Forward primer	Reverse primer
Il1b	AGG CCA CAG GTA TTT TGT	GCC CAT CCT CTG TGA CTC
Tnf	CAT CTT CTC AAA ATT CGA GTG ACA A	TGG GAG TAG ACA AGG TAC AAC CC
Il6	CTG GAT ATA ATC AGG AAA TTT GC	AAA TCT TTT ACC TCT TGG TTG A
Cxcl1	CAT GGC TGG GAT TCA CCT CA	TGA GGT GAA TCC CAG CCA TG
Actb	AGA GGG AAA TCG TGC GTG AC	CAA TAG TGA TGA CCT GGC CGT
